# Gut microbiota and its metabolites regulate insulin resistance: traditional Chinese medicine insights for T2DM

**DOI:** 10.3389/fmicb.2025.1554189

**Published:** 2025-03-19

**Authors:** Jing Liu, Fuxing Li, Le Yang, Shengping Luo, Yihui Deng

**Affiliations:** ^1^School of Integrated Chinese and Western Medicine, Hunan University of Chinese Medicine, Changsha, China; ^2^Ningxiang Traditional Chinese Medicine Hospital, Changsha, China

**Keywords:** type 2 diabetes mellitus, gut microbiota, gut microbiota metabolites, insulin resistance, traditional Chinese medicine

## Abstract

The gut microbiota is closely associated with the onset and development of type 2 diabetes mellitus (T2DM), characterized by insulin resistance (IR) and chronic low-grade inflammation. However, despite the widespread use of first-line antidiabetic drugs, IR in diabetes and its complications continue to rise. The gut microbiota and its metabolic products may promote the development of T2DM by exacerbating IR. Therefore, regulating the gut microbiota has become a promising therapeutic strategy, with particular attention given to probiotics, prebiotics, synbiotics, and fecal microbiota transplantation. This review first examines the relationship between gut microbiota and IR in T2DM, summarizing the research progress of microbiota-based therapies in modulating IR. We then delve into how gut microbiota-related metabolic products contribute to IR. Finally, we summarize the research findings on the role of traditional Chinese medicine in regulating the gut microbiota and its metabolic products to improve IR. In conclusion, the gut microbiota and its metabolic products play a crucial role in the pathophysiological process of T2DM by modulating IR, offering new insights into potential therapeutic strategies for T2DM.

## 1 Introduction

Type 2 Diabetes Mellitus (T2DM) is a metabolic disorder that accounts for 90–95% of diabetes cases and is characterized by insulin resistance (IR) (American Diabetes Association, 2015). It is associated with an increased risk of complications due to factors such as hyperglycemia, IR, low-grade inflammation, and accelerated atherosclerosis (Schlienger, 2013). The Global Burden of Disease (GBD) Study 2021, published by The Lancet, shows that in 2021, there were 529 million people worldwide living with diabetes, of which 96.0% had type 2 diabetes (T2DM). It is projected that by 2025, the prevalence of T2DM will increase from 5.9% in 2021 to 9.5%, affecting more than 1.27 billion people (GBD 2021 Diabetes Collaborators, 2023). Diabetes-related macrovascular diseases and microvascular complications, due to their high incidence, disability, and mortality rates, are significant contributors to the global health burden (Emerging Risk Factors Collaboration et al., 2010; Barrett et al., 2017). IR and chronic inflammation are key factors influencing T2DM treatment. Insulin receptors are widely distributed throughout the body, and insulin signaling primarily occurs in skeletal muscle, liver, and white adipocytes. IR is a pathological condition characterized by reduced insulin response in target tissues (mainly muscle, liver, and adipose tissue), leading to imbalances in glucose, fat, and protein metabolism (Samuel and Shulman, 2012; Petersen and Shulman, 2018).

The pathogenesis of IR in T2DM involves multiple factors, with genetics, age, gender, diet, environment, and occupation being significant risk factors (Kautzky-Willer et al., 2016; Cole and Florez, 2020; Liu et al., 2023). Recent studies have shown that the gut microbiota is a key factor in developing IR. The gut microbiota regulates signaling pathways that affect energy metabolism through the production of metabolites and interactions with the host’s intestinal cells (Lee and Lee, 2020). A reduction in gut bacterial diversity (the number or richness of bacterial species) has been associated with IR, obesity, elevated lipid levels, and increased inflammation (Le Chatelier et al., 2013). Gut microbiota also produces metabolites such as short-chain fatty acids (SCFAs), bile acids (BAs), trimethylamine N-oxide (TMAO), indole derivatives, and lipopolysaccharides (LPS), which participate in insulin signaling and induce the occurrence of T2DM through mechanisms such as IR, bile acid metabolism, lipid metabolic disorders, and endotoxemia (Gurung et al., 2020; Scheithauer et al., 2020; Zhang Y. et al., 2020; Wu et al., 2023). Modulating the gut microbiota and its metabolites can improve the effects of T2DM and underlying mechanisms (Jiang et al., 2024). Probiotics can prevent high-fat-diet (HFD) induced glucose intolerance and hyperglycemia by improving IR (Won et al., 2021). Fecal microbiota transplantation (FMT) has become an effective strategy for treating metabolic diseases, and fecal bacteria from individuals with normal blood glucose levels may represent a promising approach for treating T2DM (Zhang P. P. et al., 2020). Many herbs or their active compounds have therapeutic effects on T2DM by improving the gut microbiota structure, increasing beneficial bacteria and butyrate concentration in the gut, and inhibiting opportunistic pathogens (Xu et al., 2020; Yang X. et al., 2021). Additionally, dietary and exercise interventions can elevate bifidobacteria, improve SCFA levels, lower blood glucose, and enhance insulin sensitivity (Ojo et al., 2020; Zaharieva et al., 2020).

**Figure 1:**
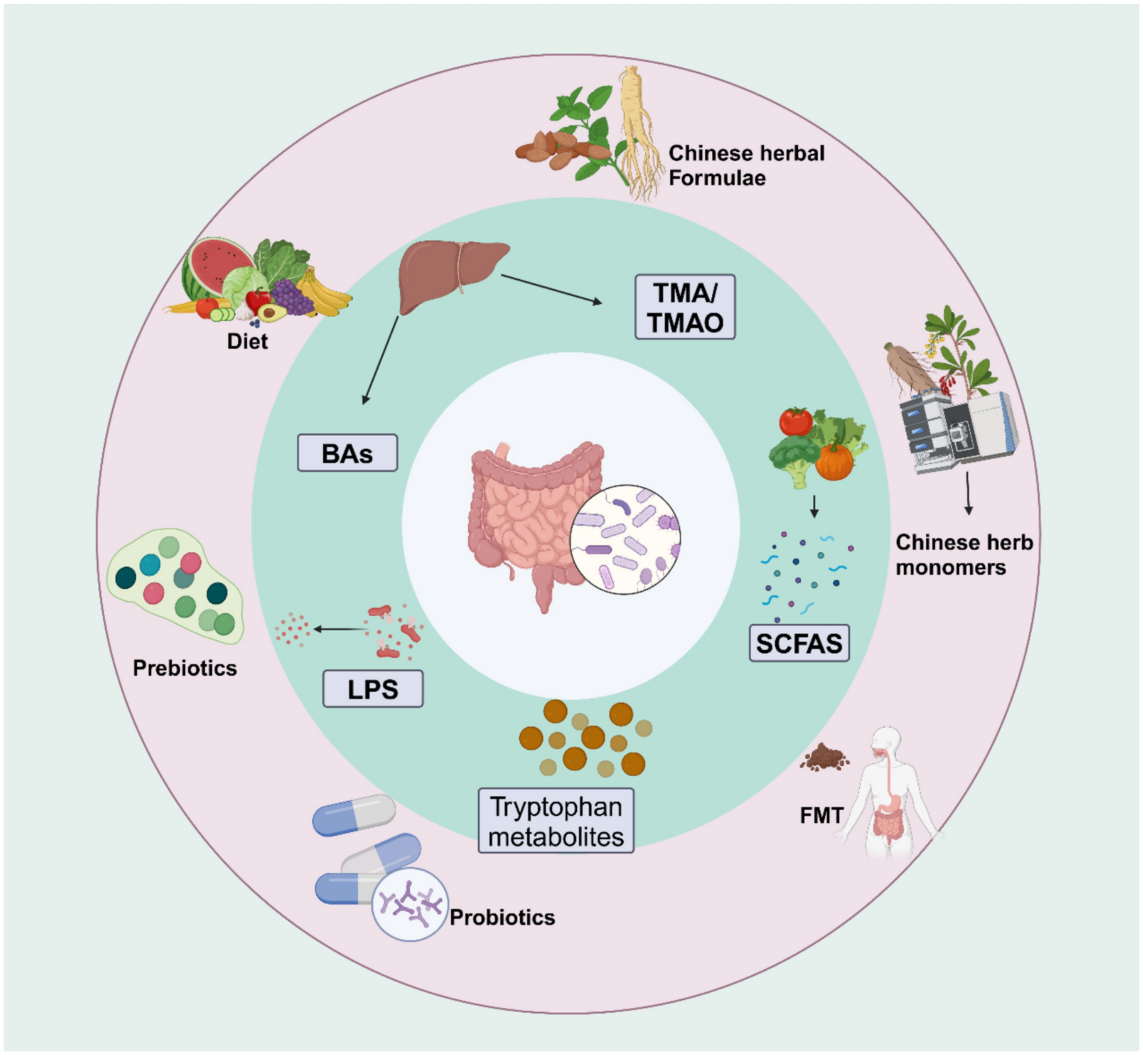
Gut microbiota metabolites and their role in improving insulin resistance: Gut microbiota metabolites such as BAs, SCFA, LPS, TMO, and tryptophan metabolites, in combination with different interventions like diet, FMT, prebiotics, probiotics, Chinese herb monomers, and Chinese herbal formulas, can regulate the gut microbiota and its metabolites to improve insulin sensitivity. Created with BioRender.com.

Therefore, investigating the role of the gut microbiota and its metabolites in developing IR could offer new strategies for preventing and treating T2DM. This approach can potentially improve IR and reduce the incidence of related metabolic and cardiovascular complications. In this review, we summarize the impact of the gut microbiota and its metabolites on the pathogenesis of IR and the mechanisms by which traditional Chinese medicine (TCM) regulates gut microbiota and its metabolites to improve IR (Figure 1).

## 2 The correlation between gut microbiota and insulin resistance

### 2.1 Mechanisms of gut dysbiosis and insulin resistance

The gut microbiota refers to the microbial community in the gastrointestinal tract, primarily consisting of bacteria, fungi, viruses, and archaea. Studies have shown that the number of gut microbiota is approximately 10 times greater than the number of human cells, with bacteria accounting for more than 90%, mainly including phyla such as Firmicutes, Bacteroidetes, Actinobacteria, and Proteobacteria (Sender et al., 2016). The composition and diversity of an individual’s gut microbiota are influenced by various factors, including diet, lifestyle, genetics, and environmental exposures, collectively shaping the microbial ecosystem. The normal gut microbiota plays a crucial role in metabolism, immune response regulation, and antimicrobial protection (Rinninella et al., 2019; Zhang, 2022). The hallmark of gut dysbiosis is the reduced diversity and abundance of bacteria and fungi, particularly those associated with functional impairments and various pathological conditions (Sun et al., 2019; Zeng et al., 2024). Extensive research has revealed a significant association between changes in the gut microbiota composition and the development of diabetes. Gut microbiota dysbiosis is characteristic of T2DM, with a reduced abundance of butyrate-producing bacteria and increased opportunistic pathogens (Qin et al., 2012). Specifically, the abundance of *Bifidobacterium* is significantly correlated with T2DM. Studies have shown a marked decrease in the total *Bifidobacterium* and *Bifidobacterium adolescentis* in diabetic groups (Xu et al., 2012). Daily supplementation of *Bifidobacterium adolescentis* restores the gut microbiota homeostasis, increases the abundance of SCFA-producing microbes, alleviates inflammation, and lowers blood glucose levels (Moroti et al., 2012; Qian et al., 2022). The presence of *Bifidobacterium, Bacteroides, Faecalibacterium, Akkermansia*, and *Roseburia* is negatively correlated with T2DM, while *Ruminococcus, Fusobacterium*, and *Blautia* show a positive correlation with T2DM (Gurung et al., 2020). Recent research has found that *Alistipes indistinctus* and *Alistipes finegoldii* are linked to IR and insulin sensitivity. These bacteria exhibit distinct carbohydrate metabolism patterns and have been shown to improve IR in mouse models by altering the host’s phenotype (Takeuchi et al., 2023). These findings underscore the critical role of microbiota composition in influencing metabolic disorders.

### 2.2 The role of microbiome therapy in T2DM

The potential for modifying the gut microbiota through dietary interventions to manage T2DM is increasingly recognized. Several beneficial bacterial genera, such as *Allobaculum*, *Bacteroides*, *Blautia*, *Butyricoccus*, and *Phascolarctobacterium*, which are characterized by SCFA production, are associated with the prevention of obesity and IR in HFD-fed rats (Zhang et al., 2012, 2015). Long-term HFD leads to T2DM and disrupts the gut microbiota, with an increase in the relative abundance of *Alistipes* and *Prevotella* and a decrease in the relative abundance of *Butyricimonas*, *Ruminococcus*, and *Bifidobacterium* (Lee et al., 2022; Zhang H. et al., 2024). High-fiber diets improve glucose homeostasis in T2DM by increasing the abundance of *Lactobacillus, Bifidobacterium*, and *Akkermansia* while decreasing the abundance of Desulfovibrio, Klebsiella and other opportunistic pathogens, but it is crucial to evaluate the long-term sustainability and practical applicability of such dietary changes across diverse populations (Chen et al., 2023; Chang et al., 2024). Furthermore, FMT has been used to demonstrate the role of gut microbiota in IR. For example, transferring the fecal microbiota of obese or IR individuals to germ-free mice results in the development of IR, while microbiota from lean, healthy individuals does not (Ridaura et al., 2013). FMT combined with lifestyle interventions or metformin has been shown to improve the gut microbiota in T2DM patients and enhance parameters such as blood lipids, IR, and body mass index (Ng et al., 2022; Wu et al., 2022). While these findings suggest a beneficial role for FMT in restoring microbiota balance and improving metabolic health, the long-term safety and efficacy of FMT require further investigation.

In addition, probiotics also hold promise as a therapeutic tool for T2DM management. *Lactobacillus rhamnosus* downregulates glucose-6-phosphatase expression, reduces fasting blood glucose, and improves glucose tolerance (Farida et al., 2020). *Lactiplantibacillus* and *Lactobacillus plantarum* inhibit intestinal enzymes and increase the concentration of hepatic antioxidant enzymes (Li et al., 2016; Lee et al., 2021; Narang et al., 2024). Several clinical trials have demonstrated that the consumption of probiotics reduces lipids and blood glucose (Tonucci et al., 2017; Hsieh et al., 2018; Mirjalili et al., 2023) and enhances glycemic management by increasing butyrate production to act as an adjunct to metformin (Palacios et al., 2020). However, the overall effectiveness of probiotics in managing T2DM may vary depending on the strain used and the individual’s baseline microbiota composition. Considering the patient’s unique microbiota profile, personalized approaches may enhance the therapeutic outcomes of probiotic interventions in T2DM management (Xiao et al., 2023). Prebiotics, which selectively promote the growth of beneficial microbes, offer an additional strategy to modulate the gut microbiome and improve insulin resistance. Resistant starch, for example, alters the selective microbiota composition to produce starch-degrading enzymes, promotes the production of gut metabolites, and enhances gut barrier function, thus preventing T2DM and obesity through the gut microbiome (Liu H. et al., 2020). Despite some promising findings, not all prebiotics, such as galacto-oligosaccharides, have significantly improved glucose and lipid metabolism, highlighting the need for more targeted prebiotic interventions (Wan et al., 2023). In conclusion, while modulation of the gut microbiota presents a promising approach for managing T2DM, it is essential to recognize that other factors, including diet, lifestyle, genetics, and environmental exposures, play critical roles in shaping the microbiota and influencing insulin resistance.

## 3 Gut microbiota metabolites regulate insulin sensitivity

IR is a key factor in the development of metabolic diseases such as T2DM, obesity, and cardiovascular diseases, involving multiple molecular mechanisms, particularly dysfunction of the insulin receptor signaling pathway, abnormalities in insulin receptor substrates (IRS), and chronic low-grade inflammation (Samuel and Shulman, 2012; Petersen and Shulman, 2018). The insulin receptor is a transmembrane receptor that, upon binding with insulin, triggers a series of intracellular signaling events through IRS. It plays a central role in mediating insulin’s effects on glucose uptake, metabolism, and cell growth (Goldfine et al., 1973; Kolterman et al., 1981). In IR, insulin receptor signaling is often impaired due to receptor defects, reduced phosphorylation, or functional changes of IRS. These defects result in weakened downstream signaling pathways, crucial for glucose transport and metabolic regulation (Kolterman et al., 1981; Schultze et al., 2012; Kearney et al., 2021). Additionally, chronic low-grade inflammation has become a critical factor in the development of IR (Shoelson et al., 2006; Olefsky and Glass, 2010). Inflammatory mediators such as tumor necrosis factor-alpha (TNF-α), interleukin-6 (IL-6), and C-reactive protein (CRP) interfere with insulin receptor signaling, further promoting the development of IR by disrupting key metabolic processes (Samokhvalov et al., 2009; Chirivi et al., 2022; Li A. et al., 2022). Understanding these potential mechanisms is crucial for identifying therapeutic targets to alleviate or reverse IR and its associated diseases.

### 3.1 Regulation of insulin receptor and substrates

#### 3.1.1 SCFAs

SCFAs, key products of gut microbiota metabolism, are the end products of fermenting indigestible food by intestinal microbes, mainly anaerobes in the cecum and colon (Macfarlane and Macfarlane, 2003). Some SCFAs are absorbed by the colon epithelium through H- or sodium-dependent monocarboxylate transporters, providing energy for colon cells (Ruppin et al., 1980). The remaining SCFAs enter circulation via the liver and portal vein, influencing the development of conditions like obesity, IR, and T2DM (Morrison and Preston, 2016). SCFAs significantly affect energy homeostasis by regulating key metabolic tissues, including adipose tissue, skeletal muscle, and the liver (Canfora et al., 2015). Studies have shown that increasing the acetate concentration in the systemic circulation may reduce lipolysis and free fatty acids (FFA) levels, thereby improving insulin sensitivity (Fernandes et al., 2012). Butyrate, as a dietary supplement, increases the phosphorylation of IRS-1 protein at Tyr632 and Akt at Thr308, preventing IR (Gao et al., 2009). meta-analysis found that different SCFA interventions reduced blood glucose in diabetic mice, with butyrate being the most effective intervention (Pham et al., 2024; Zheng et al., 2024). It can also improve AMP-activated protein kinase (AMPK) phosphorylation, increase GLP-1 secretion, and enhance insulin sensitivity (Gonzalez et al., 2019). Propionate and butyrate inhibit lipolysis and *de novo* lipogenesis, suppress acetyl-CoA carboxylase, and increase insulin-stimulated glucose uptake in primary rat adipocytes (Heimann et al., 2014). Clinical trials indicate that oral butyrate supplementation increases insulin sensitivity (Bouter et al., 2018), and long-term intake of acetate and butyrate helps improve glucose metabolism by delaying gastric emptying and intestinal absorption (Wijdeveld et al., 2023). However, some studies have found that acetate, propionate, butyrate, and mixed SCFA do not affect human blood glucose and insulin (Cherta-Murillo et al., 2022). Therefore, further studies are needed to determine the effect of SCFA on glycemic control.

The effects of SCFAs are not limited to directly influencing insulin sensitivity; they also activate specific G protein-coupled receptors (GPCRs), affecting adipocytes, immune cells, and others. Free fatty acid receptors (FFARs) belong to the GPCR family (100), with FFAR2 and FFAR3 being activated by SCFAs. This activation increases the intestinal hormones Peptide YY (PYY) and Glucagon-like peptide-1 (GLP-1), which regulate insulin signaling (Briscoe et al., 2003; Brown et al., 2003). In human and rat colon samples, SCFA receptors FFAR2 and FFAR3 are colocalized with PYY-containing enteroendocrine L cells (Karaki et al., 2008; Tazoe et al., 2009). The absence of FFAR2 and FFAR3 in pancreatic β-cells leads to increased insulin secretion and improved glucose tolerance, while FFAR2 and FFAR3 knockout mice show decreased colon PYY expression and impaired systemic glucose tolerance (Tolhurst et al., 2012; Tang et al., 2015). FFAR2 and FFAR3 mediate SCFA-induced enhancement of GLP-1 secretion. Without FFAR2 and FFAR3, SCFA-triggered GLP-1 secretion is reduced, and glucose tolerance is impaired (Tolhurst et al., 2012). SCFAs inhibit insulin signaling in adipocytes by activating FFAR2, thereby reducing fat accumulation and promoting the metabolism of lipids and glucose in other tissues (Kimura et al., 2013). Propionate and valerate activate FFAR3 to increase insulin-stimulated glucose uptake in adipocytes and skeletal muscle cells (Han et al., 2014).

#### 3.1.2 BAs

In addition to SCFAs, BAs are also considered key factors in regulating insulin sensitivity. BAs exert critical physiological functions in the intestine through microbial metabolic conversion. Primary bile acids are synthesized in the liver. In contrast, the gut microbiota produces secondary bile acids, participating in multiple metabolic processes such as fat digestion and absorption, cholesterol metabolism, and immune regulation (Wahlström et al., 2016; Guzior and Quinn, 2021). IR is positively correlated with hyperbileacidemia in diabetic populations (Sun et al., 2016), and increased total serum BAs are associated with impaired systemic insulin sensitivity, β-cell dysfunction, and elevated glucagon levels in T2DM (Wang X. H. et al., 2020). There is a relationship between elevated BA levels and impaired insulin sensitivity. BAs exert their effects by activating G protein-coupled BA receptor 5 (TGR5) and farnesoid X receptor (FXR). Studies have found that activation of TGR5 in the intestine promotes the transport of BAs, improves glucose metabolism, and enhances lipolysis and energy metabolism (Li et al., 2023). In obese mice lacking TGR5, inflammation in adipose tissue is enhanced, and insulin-stimulated AKT phosphorylation is reduced, leading to decreased insulin responsiveness in adipose tissue and exacerbating IR (Perino et al., 2014). This underscores the critical importance of TGR5 in maintaining metabolic balance.

On the other hand, FXR activation appears to have a somewhat protective effect, with studies indicating that it induces the secretion of fibroblast growth factors in the intestine, which ultimately leads to changes in BA composition. This, in turn, helps reduce obesity and insulin resistance (IR) while encouraging adipose tissue browning (Fang et al., 2015). After FXR knockout, the expression of inflammatory markers in adipose tissue macrophages and mature adipocytes decreases, protecting mice from high blood glucose and IR induced by HFD (Dehondt et al., 2023). FXR knockout mice show impaired glucose tolerance and reduced insulin sensitivity, suggesting that BA activation of FXR may improve IR by inhibiting hepatic SREBP-1c expression and/or modulating glucose-induced lipogenesis (Lefebvre et al., 2009). In sum, through their receptors TGR5 and FXR, BAs appear to exert a profound influence on glucose homeostasis, fat metabolism, and the broader metabolic landscape. As research advances, novel therapeutic approaches targeting these pathways for treating obesity, diabetes, and insulin resistance may emerge.

#### 3.1.3 TMAO

Another crucial metabolic product is trimethylamine (TMA), primarily produced by the bacterial metabolism of substrates such as phosphatidylcholine, carnitine, and betaine in the colon. TMA is oxidized in the liver by flavin monooxygenase 3 (FMO3) to form trimethylamine N-oxide (TMAO), which is associated with atherosclerosis, cholesterol reverse transport, and glucose and lipid metabolism (Koeth et al., 2013; Zhao Z. H. et al., 2019; Kong et al., 2024). One striking aspect of TMAO is its dynamic regulation of insulin sensitivity. Its levels fluctuate based on diet and an individual’s gut microbiome composition. By reducing red meat consumption and increasing plant-based foods, individuals can reduce the production of oxidized TMA and lower the risk of developing T2DM (Heianza et al., 2019; Huang et al., 2024). Higher levels of TMAO are associated with an increased risk of T2DM, possibly through effects on IR, inflammation, or lipid metabolism (Li S. Y. et al., 2022; Huang et al., 2024). The impact of TMAO on β-cell function is particularly alarming. Elevated levels of TMAO have been shown to impair glucose-stimulated insulin secretion, reduce β-cell mass, and worsen glucose tolerance, all of which can contribute to the progression of diabetes (Kong et al., 2024). In this light, TMAO’s role in the pathophysiology of diabetes becomes even more evident.

**Figure 2:**
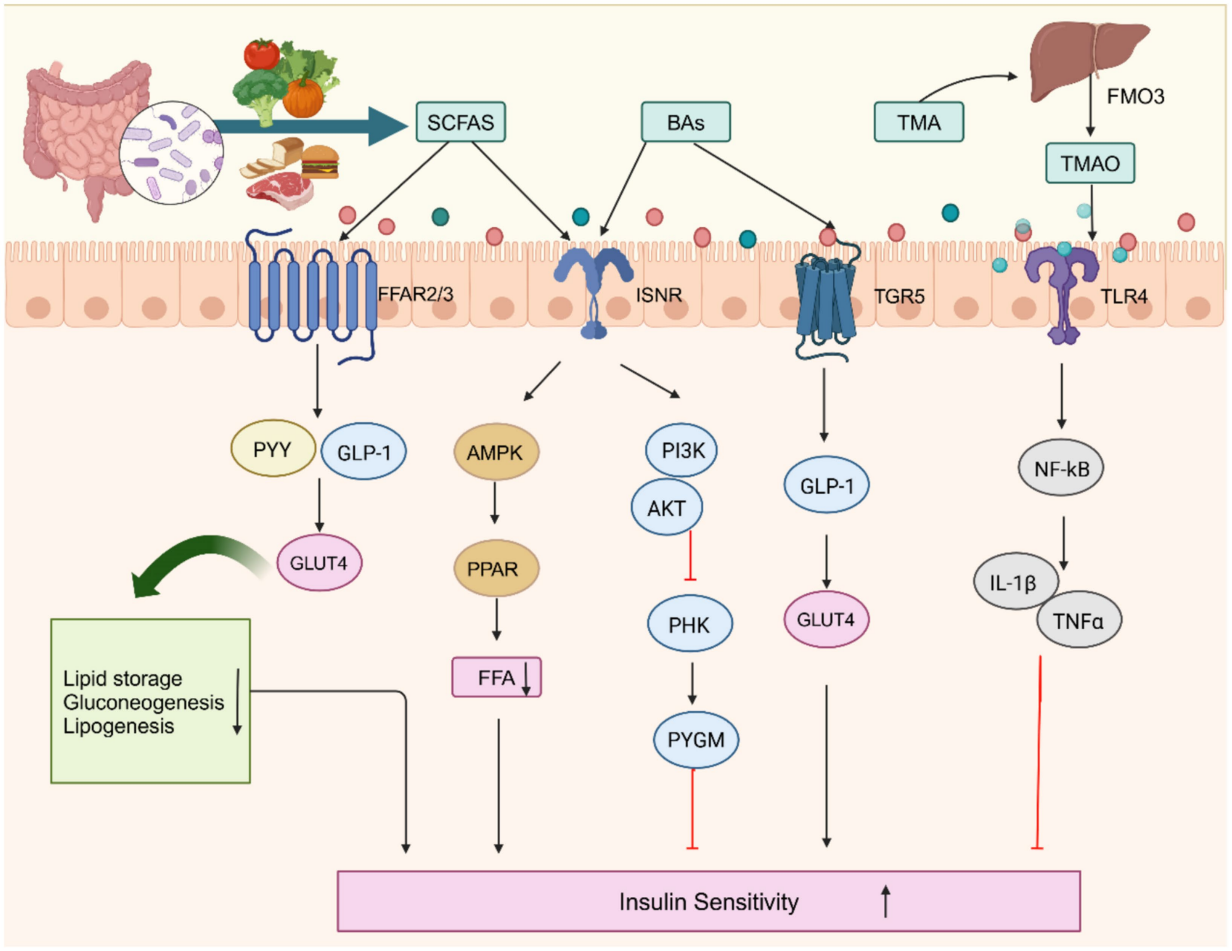
Gut microbiota metabolites regulate insulin substrates and receptors to modulate insulin resistance. ISNR, insulin receptor; GLUT4, glucose transporter 4; PHK, Phosphorylase kinase; PYGM, Glycogen phosphorylase; PPAR, peroxisome proliferator-activated; PI3K, phosphatidylinositol 3-hydroxy kinase. Created with BioRender.com.

TMAO levels are influenced by FMO3 expression. Increased liver FMO3 activity may reflect hepatic IR, as FMO3 is primarily responsible for converting TMA into TMAO (DiNicolantonio et al., 2019). FMO3 is upregulated in obese/IR male mice, and FMO3 knockdown improves glucose tolerance by reducing endoplasmic reticulum cholesterol, inducing SREBP-2, thereby inhibiting FoxO1 (Miao et al., 2015). Changes in FMO3 activity could be an important mechanism in metabolic diseases, and modulating FMO3 activity may represent a novel strategy for improving these metabolic disorders. In summary, TMAO’s role in metabolic regulation is multifaceted, with its production being influenced by diet, microbiome composition, and liver function (Figure 2).

### 3.2 Regulation of chronic low-grade inflammation

#### 3.2.1 LPS

LPS is an important component of the cell wall of Gram-negative bacteria and is known to play a significant role in IR and inflammatory responses in T2DM. Studies have shown that plasma LPS levels are positively correlated with markers of IR (Pedro et al., 2018). LPS-induced metabolic endotoxemia can lead to an increase in F4/80-positive cells in adipose tissue and an elevation in inflammatory markers, which in turn increases liver triglyceride content and exacerbates IR and fasting blood glucose levels (Cani et al., 2007). Dysbiosis induced by HFD upregulates LPS concentration, further promoting the release of pro-inflammatory cytokines such as TNF, IL-1, and IL-6, leading to systemic inflammation (Zhu et al., 2020).

LPS activates macrophages in adipose tissue and triggers the Akt–mTOR pathway. Chronic, sustained inflammation activates this pathway, ultimately leading to a decline in insulin sensitivity (Toda et al., 2020). LPS also activates the cAMP/PKA pathway or the MAPK and NF-κB signaling pathways, inhibiting IκB phosphorylation, promoting the release of free fatty acids from adipose tissue, enhancing lipolysis and inflammation, thus aggravating IR (Chung et al., 2006; Hussey et al., 2012; Jung et al., 2018a, 2018b; Chirivi et al., 2022). The end result is a vicious cycle where inflammation and insulin resistance perpetuate one another, further worsening metabolic dysfunction. Furthermore, LPS impairs insulin signaling by directly downregulating the phosphorylation of IRS, phosphoinositide 3-kinase (PI3K), and Akt, thereby reducing the responsiveness of adipocytes to insulin (Samokhvalov et al., 2009; Chirivi et al., 2022). Toll-like receptor 4 (TLR-4) is a key receptor for LPS, and LPS-mediated inflammation induced by saturated fatty acids is associated with IR via TLR-4 (González et al., 2019). Through the TLR-4 signaling pathway, LPS can activate both the MAPK and NF-κB pathways, triggering inflammatory cascades that further promote insulin resistance. Interestingly, inhibiting TLR-4 expression has been shown to improve LPS-induced IR, suggesting that targeting TLR-4 may hold therapeutic potential for managing LPS-related metabolic disorders (Kim et al., 2007; Kawamoto et al., 2008; Hussey et al., 2012). Taken together, this suggests that LPS is a key player in the development of insulin resistance and systemic inflammation, which may provide new therapeutic avenues for the management of T2DM and other metabolic disorders.

#### 3.2.2 Tryptophan metabolites

**Figure 3:**
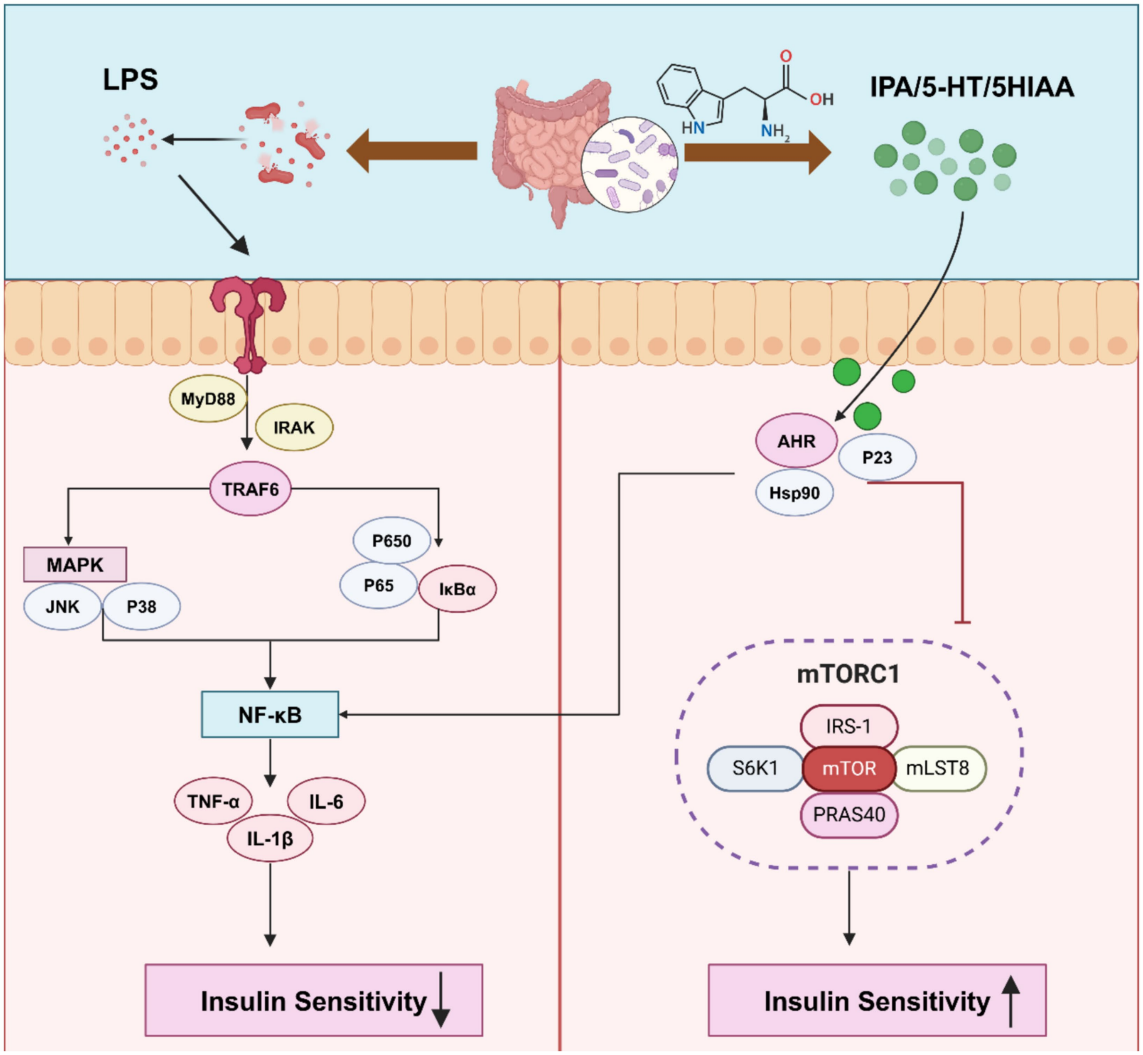
Gut microbiota metabolites participate in the regulation of chronic inflammation and insulin resistance. MyD88, myeloid differential protein-88; IRAK, interleukin receptor-associated kinases; TRAF6, tumor necrosis factor receptor-related molecules; Hsp90, heat shock protein 90; S6K1, Ribosomal protein S6 kinase; PRAS40, pras40 monoclonal antibody. Created with BioRender.com.

The gut microbiota plays a crucial role in metabolizing aromatic amino acids into metabolites such as tryptamine, indole, and other derivatives, either directly or through indirect pathways like the kynurenine and serotonin pathways (Dodd et al., 2017). Among these, tryptophan-derived metabolites, including serotonin, tryptamine, and indole, are closely related to IR and the development of T2DM (Alexeev et al., 2018). Studies have shown that tryptophan derivatives such as indole lactate are positively correlated with T2DM, while indole propionate esters are negatively correlated with T2DM (Qi et al., 2022). Additionally, tryptophan metabolites such as 5-hydroxyindole-3-acetic acid (5-HIAA) promote hepatic insulin signaling by directly activating the aryl hydrocarbon receptor (AhR), inhibiting the mTORC1 pathway, and alleviating IR induced by a high-fat diet (Du et al., 2024). Indole-3-pyruvic acid, acting through AhR, downregulates TNF-α in intestinal epithelial cells, further improving insulin sensitivity (Venkatesh et al., 2014, p. 4). Supplementing endogenous AhR ligands, such as tryptophan and indole-3-carbinol, can increase AhR expression in the gut, inhibit the expression of intercellular adhesion molecules and FMO3 in the liver, and reduce plasma levels of IL-6 and TNF-α, thus alleviating inflammation and IR (Liu W. C. et al., 2020). In contrast, AhR deficiency can prevent obesity, hepatic steatosis, IR, and inflammation induced by HFD (Xu et al., 2015). The supply of tryptophan directly affects the synthesis of serotonin (5-HT), and low levels of tryptophan suppress serotonin synthesis, which in turn impacts insulin sensitivity. As a key regulator of IR, prolonged injection of 5-HT can lead to impaired glucose tolerance and IR, indicating that changes in serotonin levels can directly affect the body’s ability to effectively use insulin, leading to IR (Liang et al., 1999; Luo et al., 1999). Serotonin triggers inflammation and adipocyte dysfunction through the serotonin reuptake transporter in adipose tissue, serotonin receptor 2B, and tryptophan hydroxylase 1, affecting insulin sensitivity (Chen et al., 2012; Yabut et al., 2020; Choi et al., 2021) (Figure 3).

## 4 Traditional Chinese medicine regulates gut microbiota and its metabolites to improve insulin resistance

T2DM is associated with dysfunction of the gut microbiota and its metabolites. The current main treatment strategies for T2DM include surgery, pharmacotherapy, exercise therapy, diet, and multifactorial approaches (Magkos et al., 2020; Su et al., 2023). Among these, insulin injection therapy is the most effective method for controlling blood glucose, but insulin injections increase the risk of cardiovascular complications (Home et al., 2014). Currently, first-line hypoglycemic drugs are the primary treatment for T2DM; however, their use is always accompanied by side effects, including weight gain, hypertension, and heart failure (Verbrugge, 2017; Davies et al., 2018). Research has shown that statins reduce blood GLP-1 levels in a microbiota-dependent manner (via the Clostridium bile acid axis), thereby worsening IR (She et al., 2024). TCM compensates for the limitations of first-line hypoglycemic drugs by reducing their side effects. *Lycium barbarum* L. increases the abundance of *Akkermansia muciniphila*, which helps alleviate liver damage by regulating the gut-liver axis (Lu et al., 2023, 2024). Clinical trials have confirmed that metformin and TCM formulas significantly improve blood glucose and lipid levels (Lu et al., 2023, 2024). However, TCM formulas have a more pronounced effect on improving insulin resistance and plasma triglyceride levels, and they have a greater impact on the gut microbiota, particularly increasing the abundance of *Blautia* spp. and *Faecalibacterium* spp., indicating that the gut microbiota is an important target for the treatment of metabolic diseases (Tong et al., 2018; Xu et al., 2022). Therefore, regulating the gut microbiota and its metabolic products to reduce the adverse risks of drug treatments represents a new therapeutic approach for T2DM. TCM’s regulation of the gut microbiota shows significant effects in the treatment of T2DM, and the gut microbiota may play a crucial role in the therapeutic effects of TCM (Wang J. et al., 2020).

### 4.1 Chinese herb monomers

The main components of Chinese herb monomers include polysaccharides, flavonoids, alkaloids, and saponins. These components regulate the abundance of gut microbiota, improve glucose metabolism, and enhance insulin sensitivity, thereby providing auxiliary treatment for diabetes (Luo et al., 2020; Bhambhani et al., 2021; Dias et al., 2021; Zhao et al., 2023). A large number of animal studies have found that TCM monomers, such as Ganoderma lucidum polysaccharides, Cyclocarya paliurus polysaccharides, and licochalcone A, can regulate the abundance of gut microbiota species (increasing *Blautia*, *Bifidobacterium*, and *Parabacteroides*, and decreasing *Aerococcus*, *Ruminococcus*, and *Enterococcus*), reduce blood sugar levels, and improve insulin resistance and glucose tolerance (Chen et al., 2020; Yao et al., 2020; Luo et al., 2023). TCM monomers not only directly regulate changes in the gut microbiota, but also improve insulin resistance by influencing the metabolic products of the gut microbiota. Coix seed polysaccharides, *Lycium barbarum* polysaccharides, and Baicalin regulate the gut microbiota composition, particularly increasing the bacteria that produce SCFAs, thereby improving abnormal glucose and lipid metabolism and showing hypoglycemic effects (Ju et al., 2019; Xia et al., 2021; Yang Y. et al., 2021). Cyclocarya paliurus polysaccharides and Astragalus membranaceus polysaccharides increase SCFA-producing bacteria, promote SCFA production, and upregulate GLP-1 and PYY expression to improve glucose tolerance (Yao et al., 2020; Song et al., 2022). Red ginseng extracts improve glucose metabolism and promote lipolysis, and energy metabolism by activating TGR5 in the gut, significantly alleviating obesity and IR (Li et al., 2023).

Animal studies have found that Baicalin can improve the balance of the gut microbiota, increase the number of bacteria-producing SCFAs, and improve glucose and lipid metabolism (Ju et al., 2019). Baicalein 7-O-glucuronide inhibits FXR-CYP7A1-mediated bile acid signaling in T2DM mice, reducing lipid accumulation in the liver and bile, thus exerting anti-diabetic effects (Yan et al., 2022). Integrating Mendelian randomization from a genetic perspective identified eight potential targets for Baicalin in the treatment of T2DM. The expression of ANPEP, BECN1, HNF1A, and ST6GAL1 increases the risk of T2DM, while the expression of PGF, RXRA, SREBF1, and USP7 lowers the risk of T2DM (Liang et al., 2024). Ginsenoside Rb1 reverses gut microbiota dysbiosis in diabetic mice, alters the levels of free fatty acids in fecal metabolites (Zhou et al., 2023), increases the abundance of *Akkermansia* spp., significantly elevates long-chain fatty acid content, improves HFD-induced dyslipidemia, and enhances insulin sensitivity (Yang X. et al., 2021; Zou et al., 2022). Clinical trials have found that diabetic patients metabolize Ginsenoside compound K slower than healthy subjects. The differences in the biotransformation ability of Ginsenoside compound K in the gut microbiota of diabetic patients and healthy subjects affect its anti-diabetic efficacy (Huang et al., 2023). The gut microbiota may enhance the efficacy of ginsenosides by influencing their biotransformation and altering the pharmacokinetics of individual ginsenosides (Kim et al., 2020, 2023). In conclusion, TCM monomers can improve insulin resistance by regulating the gut microbiota and its metabolic products (Table 1), while the gut microbiota, in turn, increases the hypoglycemic effect of these monomers by affecting their biotransformation capacity.

**Table 1:** Chinese herb monomers.

Chinese herb monomers	Research subjects	Therapeutic mechanisms and targets	References
Astragalus membranaceus	T2DM mice models	Increases SCFA-producing bacteria, promotes SCFA production, and upregulates GLP-1 and PYY expression to improve glucose tolerance	Song et al. (2022)
Baicalein 7-O-glucuronide	T2DM mice models	Inhibits FXR-CYP7A1-mediated bile acid signaling in T2DM mice, reducing lipid accumulation in the liver and bile, thus exerting anti-diabetic effects	Yan et al. (2022)
Baicalin	T2DM mice models	Increases the number of bacteria producing SCFAs and improves glucose and lipid metabolism	Ju et al. (2019)
Berberine	Zucker diabetic fatty rats	Slows the progression of prediabetes to T2DM by enhancing GLP-2, improving intestinal permeability, and modifying the gut microbiota structure	Wang et al. (2021)
Coix seed polysaccharides	T2DM mice models	Modulates gut microbial composition, especially SCFA-producing bacteria, activates the IGF1/PI3K/AKT signaling pathways, and exhibits hypoglycemic efficacy	Xia et al. (2021)
Cyclocarya paliurus polysaccharides	T2DM rat models	Increase key bacterial species that prevent diabetes, such as *Ruminococcaceae* UCG-005, and improve nutritional metabolism and energy metabolism	Li et al. (2021)
Cyclocarya paliurus polysaccharides	T2DM rat models	Increases the production of SCFAs both *in vivo* and *in vitro*, promotes the production of SCFAs and upregulating SCFA-GLP1/PYY-associated sensory mediators	Yao et al. (2020)
Ganoderma lucidum polysaccharides	T2DM rat models	Reduce the abundance of *Aerococcus*, *Ruminococcus*, *Corynebacterium*, and *Proteus*, while increasing the levels of *Blautia*, *Dehalobacterium*, *Parabacteroides*, and *Bacteroides*. These changes restore amino acid, carbohydrate, inflammation, and nucleotide metabolism to improve glucose metabolism	Chen et al. (2020)
Ginsenoside Rb1	T2DM mice models	Reverses gut microbiota dysbiosis in diabetic mice and alters the levels of free fatty acids in fecal metabolites	Zhou et al. (2023)
Ginsenoside Rb1	Obesity mice models	Increases the abundance of *Akkermansia* spp., significantly elevates long-chain fatty acid content, improves HFD-induced dyslipidemia, and enhances insulin sensitivity	Yang X. et al. (2021) and Zou et al. (2022)
Ginsenoside Ro	Obesity mice models	Promotes GLP-1 secretion and energy expenditure, improving high-fat diet-induced obesity and IR in mice by activating the TGR5 pathway	Jiang et al. (2021)
Licochalcone A	T2DM mice models	Promotes the growth of beneficial bacteria (such as *Bifidobacterium*, *Turicibacter*, *Blautia*, and *Faecococcus*) while inhibiting the growth of harmful bacteria (such as *Enterococcus*, *Dorea*, and *Arachnococcus*) and improves insulin resistance and glucose tolerance	Luo et al. (2023)
*Lycium barbarum* polysaccharides	Obesity mice models	Improve obesity by modulating the composition of intestinal flora and the metabolism of SCFAs	Yang Y. et al. (2021)
Red ginseng extracts	Obesity mice models	Improve glucose metabolism and promote lipolysis and energy metabolism by activating TGR5 in the gut, significantly alleviating obesity and IR	Li et al. (2023)

### 4.2 Chinese herbal formulae

Chinese herbal formulas are the primary prescription forms used in TCM clinical applications. Gegen Qinlian Decoction (GQD) is a widely studied anti-hyperglycemic herbal prescription. Animal studies have found that GQD regulates the gut microbiota, improves bile acid metabolism, activates the TRG5/cAMP/PKA/CREB signaling pathway, and stimulates GLP-1 secretion (Liu et al., 2024). It increases the proportion of bacteria that produce SCFAs and possess anti-inflammatory properties while decreasing the proportion of conditionally pathogenic bacteria associated with diabetes phenotypes. These effects regulate the structure of the gut microbiota, lower blood glucose levels, and reduce inflammatory cytokine levels (Tian et al., 2021). Animal experiments suggest that GQD can improve hyperglycemia and protect pancreatic function by regulating the gut microbiota and its metabolic products. Clinical trials have confirmed that, compared to metformin alone, GQD and metformin have a synergistic effect on blood glucose control (Ryuk et al., 2017; Tan et al., 2023). GQD mainly improves type 2 diabetes by increasing the abundance of *Faecalibacterium*, elevating short-chain fatty acid levels, and reducing serum inflammation-related markers, thus alleviating metabolic disorders and inflammation (Gao et al., 2024). Therefore, GQD shows potential efficacy and safety in enhancing glucose and lipid metabolism and alleviating insulin resistance, making it a promising supplementary therapy for T2DM.

Shenzhu tiaopi granule (SZTP) can increase the relative abundance of *Lactobacillus* in the intestines of T2DM rats, reduce the relative abundance of *Allobaculum* and *Desulfovibrionaceae*, improve blood glucose and lipid levels in T2DM rats (Zhao J. et al., 2019), decrease LPS and IL-1β levels, increase the abundance of *Intestinimonas*, reduce the abundance of *Eubacterium coprostanoligenes*, regulate bile acid biosynthesis and cholesterol metabolism, and alleviate hyperglycemia (Zhao and Fang, 2024). Clinical studies have confirmed that SZTP, combined with lifestyle interventions, reduces the conversion rate from impaired glucose tolerance (IGT) to diabetes and increases the conversion rate from IGT to normal blood glucose levels (Fang et al., 2014). JinQi Jiangtang Tablet (JQJT) increases *Akkermansia*, decreases *Desulfovibrio*, increases the concentrations of acetate, propionate, and butyrate, enhances intestinal barrier function, reduces host inflammation, improves insulin resistance in T2DM, regulates gut microbiota, and promotes SCFA production (Cao et al., 2019). Clinical trials have found that after JQJT intervention, the risk of progression from prediabetes to diabetes was 0.58 times lower than in the placebo group, and the likelihood of reaching normal blood glucose levels was 1.41 times higher than in the placebo group. After 12 months of intervention, the percentage of patients with normalized blood glucose was 41.8%, compared to 27.8% in the control group (Wang et al., 2017). Chinese Herbal Formulae Tianqi treatment for 12 months reduced diabetes risk by 32.1%, and no serious adverse events occurred in the trial (Lian et al., 2014). Qinglian Hongqu decoction and JiangTang Sanhuang pill (JTSH) activate the FXR/FGF15 and TGR5/GLP-1 signaling pathways in the gut, reducing lipid accumulation and insulin resistance (Tawulie et al., 2023; Zhang Z. et al., 2024). A retrospective cohort study found that 1-year treatment with JTSH tablets reduced the risk of poor glycemic control by 17.00%. T2DM patients were satisfied with the anti-diabetic effect of JTSH tablets, which significantly lowered blood glucose and insulin resistance and improved pancreatic beta-cell function (Shao et al., 2022). The above animal experiments and clinical trials suggest that Chinese Herbal Formula can significantly reduce the incidence of T2DM in subjects with impaired glucose tolerance, making it an effective intervention for preventing and treating type 2 diabetes (Table 2).

**Table 2:** Chinese herbal formulae.

Chinese herbal formulae	Research subjects	Therapeutic mechanisms and targets	References
Baihu Rensheng decoction	T2DM rat models	Increases the relative abundance of *Lactobacillus*, *Blautia*, and *Anaerostipes* in the gut of T2DM rats while decreasing the relative abundance of *Allobaculum*, *Candidatus Saccharimonas*, and *Ruminococcus*. It inhibits TLR4/NF-κB-mediated inflammation and alleviates hyperglycemia and inflammatory responses	Yao et al. (2022)
Gegen Qinlian decoction	T2DM mice models	Regulates the gut microbiota, improves bile acid metabolism, activates the TRG5/cAMP/PKA/CREB signaling pathway, stimulates GLP-1 secretion, and significantly reduces blood glucose levels in T2DM mice, improving oral glucose tolerance	Liu et al. (2024)
Gegen Qinlian decoction	T2DM rat models	Regulates the structure of the gut microbiome by increasing the proportion of SCFA-producing and anti-inflammatory bacteria while decreasing the proportion of conditionally pathogenic bacteria associated with diabetic phenotypes, which helps lower blood glucose and inflammatory cytokine levels	Tian et al. (2021)
Gegen Qinlian decoction	patients with T2DM and T2DM mice models	Improves T2DM by increasing the abundance of *Faecalibacterium*, elevating SCFA levels, and reducing serum inflammation-related markers, thereby alleviating metabolic disorders and inflammatory states	Gao et al. (2024)
JiangTang Sanhuang pill	T2DM rat models	Activate the FXR/FGF15 and TGR5/GLP-1 signaling pathways in the gut, reducing lipid accumulation and insulin resistance	Tawulie et al. (2023)
JiangTang Sanhuang pill	patients with T2DM	Reasonable blood glucose control may positively correlate with the duration of JTSH tablet administration. Patients with T2DM were satisfied with the Anti-diabetic effects of JTSH tablets, which can significantly reduce blood glucose and insulin resistance and improve the function of islet cells	Shao et al. (2022)
JinQi Jiangtang Tablet	T2DM mice models	Increases *Akkermansia*, decreases *Desulfovibrio*, increases the concentrations of acetate, propionate, and butyrate, enhances intestinal barrier function, reduces host inflammation, improves insulin resistance in T2DM, regulates gut microbiota, and promotes SCFA production	Cao et al. (2019)
JinQi Jiangtang Tablet	patients with pre-diabetes	The incidence of diabetes upon treatment completion was 16.5% in the JQJT tablets group compared with 28.9% in the control group. The percentage of patients with normalized blood glucose upon 12-month intervention was 41.8% in the JQJT tablets group compared with 27.8% in the control group	Wang et al. (2017)
PuRenDan	T2DM rat models	Reduces serum lipid metabolism biomarkers and inflammatory factors, regulates the levels of pantothenic acid, 1-methylhistamine, and 1-methylhistidine, participates in the biosynthesis of pantothenic acid and coenzyme A, histidine metabolism, and secondary bile acid biosynthesis, thus improving blood glucose and IR	Ma et al. (2024)
Shenzhu Tiaopi Granule	T2DM rat models	Increase the relative abundance of Lactobacillus in the intestines of T2DM rats, reduce the relative abundance of Allobaculum and Desulfovibrionaceae, and improve blood glucose and lipid levels in T2DM rats	Zhao J. et al. (2019)
ShenZhu TiaoPi Granule	T2DM rat models	Decrease LPS and IL-1β levels, increase the abundance of Intestinimonas, reduce the abundance of *Eubacterium coprostanoligenes*, regulate bile acid biosynthesis and cholesterol metabolism, and alleviate hyperglycemia	Zhao and Fang (2024)
ShenZhu TiaoPi Granule	patients with impaired glucose tolerance	Reduces the conversion rate from impaired glucose tolerance (IGT) to diabetes and increases the conversion rate from IGT to normal blood glucose levels	Fang et al. (2014)
Shengmai San Formula	Obesity mice models	Reduces the abundance of lactobacilli carrying bile salt hydrolase, increases TCA content, promotes M2 macrophage polarization in adipose tissue, and enhances Slit3 release, improving glucose and lipid metabolism	Wang et al. (2024)
Tianqi	patients with impaired glucose tolerance	Reduced diabetes risk by 32.1% and no serious adverse events occurred in the trial	Lian et al. (2014)

## 5 Limitations and research prospects of TCM treatment

Specific TCM monomers have progressed in regulating gut microbiota to treat diabetes. For example, baicalin and ginsenosides can improve gut microbiota structure, enhance insulin sensitivity, and regulate glucose and lipid metabolism (Zheng et al., 2019; Sun et al., 2021). However, research has mainly focused on animal experiments and preliminary clinical trials, and clinical translation still faces challenges significantly since the diversity and complexity of gut microecology may vary considerably between humans and animals (Amato, 2016; Cao et al., 2020). The biotransformation capabilities of different TCM monomers and the mechanisms of their metabolites are not yet precise, and further research is needed on the differences in individual responses to gut microbiota, as well as the relationship between drug dosage and effectiveness (Kim et al., 2020; Deng et al., 2024). Although TCM formulas can regulate gut microbiota and improve diabetes symptoms, their molecular mechanisms still require in-depth exploration. In addition, TCM formulas are complex in composition, and extracting practical components precisely and optimizing dosages and treatment protocols remains a challenge. In the future, it is essential to identify the targets of TCM monomers, optimize biotransformation pathways, explore individualized treatment plans, and conduct large-scale, multi-center, long-term randomized controlled trials to verify efficacy and safety, as well as assess their potential in different diabetes subtypes.

## 6 Conclusions and future perspectives

In summary, the gut microbiota and its metabolites are crucial in the onset and progression of IR in T2DM. SCFAs, BAs, TMAO, LPS, and indole derivatives significantly regulate glucose metabolism by improving insulin sensitivity, promoting gut hormone secretion, and inhibiting inflammation. TCM formulas and individual herbs, such as *Lycium barbarum*, Ganoderma lucidum, and Baicalin, help improve glucose metabolism and insulin sensitivity by promoting beneficial gut bacteria. Furthermore, TCM monomers, such as polysaccharides, flavonoids, and alkaloids, show the potential to directly influence gut microbiota and metabolic products, highlighting their significant clinical efficacy in managing T2DM. These findings highlight the key role of gut microbial metabolites in IR and contribute to exploring new therapies for metabolic diseases. They help understand how the gut microbiota and its metabolites regulate IR and provide new therapeutic targets for clinical treatment.

Although significant progress has been made in current research, many issues remain unresolved, such as the variability of microbiota among individuals, further clarification of the specific mechanisms, and the feasibility of clinical applications. Future studies should focus on exploring the individual differences in the metabolic effects of different microbiota communities and conduct clinical trials to assess the clinical application value of microbiota-based therapies in preventing and treating T2DM. Attention should also be given to optimizing the use of TCM in this field and its potential for combination therapy with conventional drugs. These studies offer new approaches to diabetes treatment, primarily through strategies regulating gut microbiota and its metabolites. TCM and related complementary therapies are expected to become effective adjuncts in treating T2DM, improving blood glucose control, and reducing drug side effects.

## References

[ref1] Alexeev E. E. Lanis J. M. Kao D. J. Campbell E. L. Kelly C. J. Battista K. D.. (2018). Microbiota-derived indole metabolites promote human and murine intestinal homeostasis through regulation of Interleukin-10 receptor. Am. J. Pathol. 188, 1183–1194. doi: 10.1016/j.ajpath.2018.01.011, PMID: 29454749 PMC5906738

[ref2] Amato K. R. (2016). Incorporating the gut microbiota into models of human and non-human primate ecology and evolution. Am. J. Phys. Anthropol. 159, S196–S215. doi: 10.1002/ajpa.22908, PMID: 26808106

[ref3] American Diabetes Association (2015). (2) classification and diagnosis of diabetes. Diabetes Care 38, S8–S16. doi: 10.2337/dc15-S005 25537714

[ref4] Barrett E. J. Liu Z. Khamaisi M. King G. L. Klein R. Klein B. E. K.. (2017). Diabetic microvascular disease: An Endocrine Society scientific statement. J. Clin. Endocrinol. Metab. 102, 4343–4410. doi: 10.1210/jc.2017-01922, PMID: 29126250 PMC5718697

[ref5] Bhambhani S. Kondhare K. R. Giri A. P. (2021). Diversity in chemical structures and biological properties of plant alkaloids. Molecules 26:3374. doi: 10.3390/molecules26113374, PMID: 34204857 PMC8199754

[ref6] Bouter K. Bakker G. Levin E. Hartstra A. Kootte R. Udayappan S.. (2018). Differential metabolic effects of oral butyrate treatment in lean versus metabolic syndrome subjects. Clin. Transl. Gastroenterol. 9:155. doi: 10.1038/s41424-018-0025-4, PMID: 29799027 PMC5968024

[ref7] Briscoe C. P. Tadayyon M. Andrews J. L. Benson W. G. Chambers J. K. Eilert M. M.. (2003). The orphan G protein-coupled receptor GPR40 is activated by medium and long chain fatty acids. J. Biol. Chem. 278, 11303–11311. doi: 10.1074/jbc.M211495200, PMID: 12496284

[ref8] Brown A. J. Goldsworthy S. M. Barnes A. A. Eilert M. M. Tcheang L. Daniels D.. (2003). The orphan G protein-coupled receptors GPR41 and GPR43 are activated by propionate and other short chain carboxylic acids. J. Biol. Chem. 278, 11312–11319. doi: 10.1074/jbc.M211609200 12496283

[ref9] Canfora E. E. Jocken J. W. Blaak E. E. (2015). Short-chain fatty acids in control of body weight and insulin sensitivity. Nat. Rev. Endocrinol. 11, 577–591. doi: 10.1038/nrendo.2015.128, PMID: 26260141

[ref10] Cani P. D. Amar J. Iglesias M. A. Poggi M. Knauf C. Bastelica D.. (2007). Metabolic endotoxemia initiates obesity and insulin resistance. Diabetes 56, 1761–1772. doi: 10.2337/db06-1491 17456850

[ref11] Cao T. T. B. Wu K.-C. Hsu J.-L. Chang C.-S. Chou C. Lin C.-Y.. (2020). Effects of non-insulin anti-hyperglycemic agents on gut microbiota: a systematic review on human and animal studies. Front. Endocrinol. 11:573891. doi: 10.3389/fendo.2020.573891, PMID: 33071980 PMC7538596

[ref12] Cao Y. Yao G. Sheng Y. Yang L. Wang Z. Yang Z.. (2019). JinQi Jiangtang tablet regulates gut microbiota and improve insulin sensitivity in type 2 diabetes mice. J. Diabetes Res. 2019, 1872134–1872112. doi: 10.1155/2019/1872134, PMID: 30733971 PMC6348821

[ref13] Chang W.-L. Chen Y.-E. Tseng H.-T. Cheng C.-F. Wu J.-H. Hou Y.-C. (2024). Gut microbiota in patients with prediabetes. Nutrients 16:1105. doi: 10.3390/nu16081105, PMID: 38674796 PMC11053759

[ref14] Chen L. Liu B. Ren L. Du H. Fei C. Qian C.. (2023). High-fiber diet ameliorates gut microbiota, serum metabolism and emotional mood in type 2 diabetes patients. Front. Cell. Infect. Microbiol. 13:1069954. doi: 10.3389/fcimb.2023.1069954, PMID: 36794003 PMC9922700

[ref15] Chen X. Margolis K. J. Gershon M. D. Schwartz G. J. Sze J. Y. (2012). Reduced serotonin reuptake transporter (SERT) function causes insulin resistance and hepatic steatosis independent of food intake. PLoS One 7:e32511. doi: 10.1371/journal.pone.0032511, PMID: 22412882 PMC3297606

[ref16] Chen M. Xiao D. Liu W. Song Y. Zou B. Li L.. (2020). Intake of Ganoderma lucidum polysaccharides reverses the disturbed gut microbiota and metabolism in type 2 diabetic rats. Int. J. Biol. Macromol. 155, 890–902. doi: 10.1016/j.ijbiomac.2019.11.047, PMID: 31712153

[ref17] Cherta-Murillo A. Pugh J. E. Alaraj-Alshehhi S. Hajjar D. Chambers E. S. Frost G. S. (2022). The effects of SCFAs on glycemic control in humans: a systematic review and meta-analysis. Am. J. Clin. Nutr. 116, 335–361. doi: 10.1093/ajcn/nqac085, PMID: 35388874 PMC9348993

[ref18] Chirivi M. Rendon C. J. Myers M. N. Prom C. M. Roy S. Sen A.. (2022). Lipopolysaccharide induces lipolysis and insulin resistance in adipose tissue from dairy cows. J. Dairy Sci. 105, 842–855. doi: 10.3168/jds.2021-20855, PMID: 34696909

[ref19] Choi W. G. Choi W. Oh T. J. Cha H.-N. Hwang I. Lee Y. K.. (2021). Inhibiting serotonin signaling through HTR2B in visceral adipose tissue improves obesity-related insulin resistance. J. Clin. Invest. 131:e145331. doi: 10.1172/JCI145331, PMID: 34618686 PMC8631597

[ref20] Chung S. Lapoint K. Martinez K. Kennedy A. Boysen Sandberg M. McIntosh M. K. (2006). Preadipocytes mediate lipopolysaccharide-induced inflammation and insulin resistance in primary cultures of newly differentiated human adipocytes. Endocrinology 147, 5340–5351. doi: 10.1210/en.2006-0536, PMID: 16873530

[ref21] Cole J. B. Florez J. C. (2020). Genetics of diabetes and diabetes complications. Nat. Rev. Nephrol. 16, 377–390. doi: 10.1038/s41581-020-0278-5, PMID: 32398868 PMC9639302

[ref22] Davies M. J. D’Alessio D. A. Fradkin J. Kernan W. N. Mathieu C. Mingrone G.. (2018). Management of Hyperglycemia in type 2 diabetes, 2018. A consensus report by the American Diabetes Association (ADA) and the European Association for the Study of diabetes (EASD). Diabetes Care 41, 2669–2701. doi: 10.2337/dci18-0033, PMID: 30291106 PMC6245208

[ref23] Dehondt H. Marino A. Butruille L. Mogilenko D. A. Nzoussi Loubota A. C. Chávez-Talavera O.. (2023). Adipocyte-specific FXR-deficiency protects adipose tissue from oxidative stress and insulin resistance and improves glucose homeostasis. Mol. Metab. 69:101686. doi: 10.1016/j.molmet.2023.101686, PMID: 36746333 PMC9958065

[ref24] Deng M.-S. Huang S.-T.-Z. Xu Y.-N. Shao L. Wang Z.-G. Chen L.-J.. (2024). *In vivo* pharmacokinetics of ginsenoside compound K mediated by gut microbiota. PLoS One 19:e0307286. doi: 10.1371/journal.pone.0307286, PMID: 39178246 PMC11343376

[ref25] Dias M. C. Pinto D. C. G. A. Silva A. M. S. (2021). Plant flavonoids: chemical characteristics and biological activity. Molecules 26:5377. doi: 10.3390/molecules26175377, PMID: 34500810 PMC8434187

[ref26] DiNicolantonio J. J. McCarty M. OKeefe J. (2019). Association of moderately elevated trimethylamine N-oxide with cardiovascular risk: is TMAO serving as a marker for hepatic insulin resistance. Open Heart 6:e000890. doi: 10.1136/openhrt-2018-000890, PMID: 30997120 PMC6443140

[ref27] Dodd D. Spitzer M. H. Van Treuren W. Merrill B. D. Hryckowian A. J. Higginbottom S. K.. (2017). A gut bacterial pathway metabolizes aromatic amino acids into nine circulating metabolites. Nature 551, 648–652. doi: 10.1038/nature24661, PMID: 29168502 PMC5850949

[ref28] Du W. Jiang S. Yin S. Wang R. Zhang C. Yin B.-C.. (2024). The microbiota-dependent tryptophan metabolite alleviates high-fat diet-induced insulin resistance through the hepatic AhR/TSC2/mTORC1 axis. Proc. Natl. Acad. Sci. USA 121:e2400385121. doi: 10.1073/pnas.2400385121, PMID: 39167602 PMC11363250

[ref29] Emerging Risk Factors Collaboration Sarwar N. Gao P. Seshasai S. R. K. Gobin R. Kaptoge S.. (2010). Diabetes mellitus, fasting blood glucose concentration, and risk of vascular disease: a collaborative meta-analysis of 102 prospective studies. Lancet 375, 2215–2222. doi: 10.1016/S0140-6736(10)60484-9, PMID: 20609967 PMC2904878

[ref30] Fang S. Suh J. M. Reilly S. M. Yu E. Osborn O. Lackey D.. (2015). Intestinal FXR agonism promotes adipose tissue browning and reduces obesity and insulin resistance. Nat. Med. 21, 159–165. doi: 10.1038/nm.3760, PMID: 25559344 PMC4320010

[ref31] Fang Z. Zhao J. Shi G. Shu Y. Ni Y. Wang H.. (2014). Shenzhu Tiaopi granule combined with lifestyle intervention therapy for impaired glucose tolerance: a randomized controlled trial. Complement. Ther. Med. 22, 842–850. doi: 10.1016/j.ctim.2014.08.004, PMID: 25440374

[ref32] Farida E. Nuraida L. Giriwono P. E. Jenie B. S. L. (2020). *Lactobacillus rhamnosus* reduces blood glucose level through downregulation of gluconeogenesis gene expression in Streptozotocin-induced diabetic rats. Int. J. Food Sci. 2020:6108575. doi: 10.1155/2020/6108575, PMID: 32399477 PMC7201496

[ref33] Fernandes J. Vogt J. Wolever T. M. (2012). Intravenous acetate elicits a greater free fatty acid rebound in Normal than Hyperinsulinaemic humans. Eur. J. Clin. Nutr. 66, 1029–1034. doi: 10.1038/ejcn.2012.98, PMID: 22828730 PMC3937122

[ref34] Gao Z. Yin J. Zhang J. Ward R. E. Martin R. J. Lefevre M.. (2009). Butyrate improves insulin sensitivity and increases energy expenditure in mice. Diabetes 58, 1509–1517. doi: 10.2337/db08-1637, PMID: 19366864 PMC2699871

[ref35] Gao Z. Zhang W. He L. Wang H. Li Y. Jiang X.. (2024). Double-blinded, randomized clinical trial of Gegen Qinlian decoction pinpoints Faecalibacterium as key gut bacteria in alleviating hyperglycemia. Precis. Clin. Med. 7:pbae003. doi: 10.1093/pcmedi/pbae003, PMID: 38495337 PMC10941319

[ref36] GBD 2021 Diabetes Collaborators (2023). Global, regional, and national burden of diabetes from 1990 to 2021, with projections of prevalence to 2050: a systematic analysis for the global burden of disease study 2021. Lancet 402, 203–234. doi: 10.1016/S0140-6736(23)01301-6, PMID: 37356446 PMC10364581

[ref37] Goldfine I. D. Kahn C. R. Neville D. M. Roth J. Garrison M. M. Bates R. W. (1973). Decreased binding of insulin to its receptors in rats with hormone induced insulin resistance. Biochem. Biophys. Res. Commun. 53, 852–857. doi: 10.1016/0006-291x(73)90171-x 4354452

[ref38] González F. Considine R. V. Abdelhadi O. A. Acton A. J. (2019). Saturated fat ingestion promotes lipopolysaccharide-mediated inflammation and insulin resistance in polycystic ovary syndrome. J. Clin. Endocrinol. Metab. 104, 934–946. doi: 10.1210/jc.2018-01143, PMID: 30590569 PMC6364509

[ref39] Gonzalez A. Krieg R. Massey H. D. Carl D. Ghosh S. Gehr T. W. B.. (2019). Sodium butyrate ameliorates insulin resistance and renal failure in CKD rats by modulating intestinal permeability and mucin expression. Nephrol. Dial. Transplant. 34, 783–794. doi: 10.1093/ndt/gfy238, PMID: 30085297 PMC6503301

[ref40] Gurung M. Li Z. You H. Rodrigues R. Jump D. B. Morgun A.. (2020). Role of gut microbiota in type 2 diabetes pathophysiology. EBioMedicine 51:102590. doi: 10.1016/j.ebiom.2019.11.051, PMID: 31901868 PMC6948163

[ref41] Guzior D. V. Quinn R. A. (2021). Review: microbial transformations of human bile acids. Microbiome 9:140. doi: 10.1186/s40168-021-01101-1, PMID: 34127070 PMC8204491

[ref42] Han J.-H. Kim I.-S. Jung S.-H. Lee S.-G. Son H.-Y. Myung C.-S. (2014). The effects of propionate and valerate on insulin responsiveness for glucose uptake in 3T3-L1 adipocytes and C2C12 myotubes via G protein-coupled receptor 41. PLoS One 9:e95268. doi: 10.1371/journal.pone.0095268, PMID: 24748202 PMC3991595

[ref44] Heianza Y. Sun D. Li X. DiDonato J. A. Bray G. A. Sacks F. M.. (2019). Gut microbiota metabolites, amino acid metabolites and improvements in insulin sensitivity and glucose metabolism: the POUNDS lost trial. Gut 68, 263–270. doi: 10.1136/gutjnl-2018-316155, PMID: 29860242 PMC6275143

[ref45] Heimann E. Nyman M. Degerman E. (2014). Propionic acid and butyric acid inhibit lipolysis and de novo lipogenesis and increase insulin-stimulated glucose uptake in primary rat adipocytes. Adipocytes 4, 81–88. doi: 10.4161/21623945.2014.960694, PMID: 26167409 PMC4496978

[ref46] Home P. Riddle M. Cefalu W. T. Bailey C. J. Bretzel R. G. Del Prato S.. (2014). Insulin therapy in people with type 2 diabetes: opportunities and challenges? Diabetes Care 37, 1499–1508. doi: 10.2337/dc13-2743, PMID: 24855154 PMC5131884

[ref9001] Hsieh M.-C. Tsai W.-H. Jheng Y.-P. Su S.-L. Wang S.-Y. Lin C.-C.. (2018). The beneficial effects of Lactobacillus reuteri ADR-1 or ADR-3 consumption on type 2 diabetes mellitus: a randomized, double-blinded, placebo-controlled trial. Sci. Rep. 8:16791. doi: 10.1038/s41598-018-35014-1 30429496 PMC6235926

[ref47] Huang S. Shao L. Chen M. Wang L. Liu J. Zhang W.. (2023). Biotransformation differences of ginsenoside compound K mediated by the gut microbiota from diabetic patients and healthy subjects. Chin. J. Nat. Med. 21, 723–729. doi: 10.1016/S1875-5364(23)60402-9, PMID: 37879791

[ref48] Huang Y. Wu Y. Zhang Y. Bai H. Peng R. Ruan W.. (2024). Dynamic changes in gut microbiota-derived metabolite trimethylamine-N-oxide and risk of type 2 diabetes mellitus: potential for dietary changes in diabetes prevention. Nutrients 16:1711. doi: 10.3390/nu16111711, PMID: 38892643 PMC11174887

[ref49] Hussey S. E. Liang H. Costford S. R. Klip A. DeFronzo R. A. Sanchez-Avila A.. (2012). TAK-242, a small-molecule inhibitor of toll-like receptor 4 signalling, unveils similarities and differences in lipopolysaccharide- and lipid-induced inflammation and insulin resistance in muscle cells. Biosci. Rep. 33, 37–47. doi: 10.1042/BSR20120098, PMID: 23050932 PMC3522475

[ref50] Jiang R. Cong Z. Zheng L. Zhang L. Guan Q. Wang S.. (2024). Global research trends in regulating gut microbiome to improve type 2 diabetes mellitus: bibliometrics and visual analysis. Front. Endocrinol. 15:1401070. doi: 10.3389/fendo.2024.1401070, PMID: 38887274 PMC11181692

[ref51] Jiang L.-S. Li W. Zhuang T.-X. Yu J.-J. Sun S. Ju Z.-C.. (2021). Ginsenoside Ro ameliorates high-fat diet-induced obesity and insulin resistance in mice via activation of the G protein-coupled bile acid receptor 5 pathway. J. Pharmacol. Exp. Ther. 377, 441–451. doi: 10.1124/jpet.120.000435, PMID: 33820830

[ref52] Ju M. Liu Y. Li M. Cheng M. Zhang Y. Deng G.. (2019). Baicalin improves intestinal microecology and abnormal metabolism induced by high-fat diet. Eur. J. Pharmacol. 857:172457. doi: 10.1016/j.ejphar.2019.172457, PMID: 31202804

[ref53] Jung T. W. Chung Y. H. Kim H.-C. Abd El-Aty A. M. Jeong J. H. (2018a). Protectin DX attenuates LPS-induced inflammation and insulin resistance in adipocytes via AMPK-mediated suppression of the NF-κB pathway. Am. J. Physiol. Endocrinol. Metab. 315, E543–E551. doi: 10.1152/ajpendo.00408.2017, PMID: 29584445

[ref54] Jung T. W. Park H. S. Choi G. H. Kim D. Lee T. (2018b). β-Aminoisobutyric acid attenuates LPS-induced inflammation and insulin resistance in adipocytes through AMPK-mediated pathway. J. Biomed. Sci. 25:27. doi: 10.1186/s12929-018-0431-7, PMID: 29592806 PMC5875012

[ref55] Karaki S.-I. Tazoe H. Hayashi H. Kashiwabara H. Tooyama K. Suzuki Y.. (2008). Expression of the short-chain fatty acid receptor, GPR43, in the human colon. J. Mol. Histol. 39, 135–142. doi: 10.1007/s10735-007-9145-y 17899402

[ref56] Kautzky-Willer A. Harreiter J. Pacini G. (2016). Sex and gender differences in risk, pathophysiology and complications of type 2 diabetes mellitus. Endocr. Rev. 37, 278–316. doi: 10.1210/er.2015-1137, PMID: 27159875 PMC4890267

[ref57] Kawamoto T. Ii M. Kitazaki T. Iizawa Y. Kimura H. (2008). TAK-242 selectively suppresses toll-like receptor 4-signaling mediated by the intracellular domain. Eur. J. Pharmacol. 584, 40–48. doi: 10.1016/j.ejphar.2008.01.026, PMID: 18299127

[ref58] Kearney A. L. Norris D. M. Ghomlaghi M. Lok K. Wong M. Humphrey S. J.. (2021). Akt phosphorylates insulin receptor substrate to limit PI3K-mediated PIP3 synthesis. eLife 10:e66942. doi: 10.7554/eLife.66942, PMID: 34253290 PMC8277355

[ref59] Kim J.-K. Choi M. S. Jeung W. Ra J. Yoo H. H. Kim D.-H. (2020). Effects of gut microbiota on the pharmacokinetics of protopanaxadiol ginsenosides Rd, Rg3, F2, and compound K in healthy volunteers treated orally with red ginseng. J. Ginseng Res. 44, 611–618. doi: 10.1016/j.jgr.2019.05.012, PMID: 32617041 PMC7322745

[ref60] Kim J.-K. Choi M. S. Park H.-S. Kee K. H. Kim D.-H. Yoo H. H. (2023). Pharmacokinetic profiling of Ginsenosides, Rb1, Rd, and Rg3, in mice with antibiotic-induced gut microbiota alterations: implications for variability in the therapeutic efficacy of red ginseng extracts. Foods 12:4342. doi: 10.3390/foods12234342, PMID: 38231867 PMC10706259

[ref61] Kim F. Pham M. Luttrell I. Bannerman D. D. Tupper J. Thaler J.. (2007). Toll-like receptor-4 mediates vascular inflammation and insulin resistance in diet-induced obesity. Circ. Res. 100, 1589–1596. doi: 10.1161/CIRCRESAHA.106.142851, PMID: 17478729

[ref62] Kimura I. Ozawa K. Inoue D. Imamura T. Kimura K. Maeda T.. (2013). The gut microbiota suppresses insulin-mediated fat accumulation via the short-chain fatty acid receptor GPR43. Nat. Commun. 4:1829. doi: 10.1038/ncomms2852 23652017 PMC3674247

[ref63] Koeth R. A. Wang Z. Levison B. S. Buffa J. A. Org E. Sheehy B. T.. (2013). Intestinal microbiota metabolism of L-carnitine, a nutrient in red meat, promotes atherosclerosis. Nat. Med. 19, 576–585. doi: 10.1038/nm.3145, PMID: 23563705 PMC3650111

[ref64] Kolterman O. G. Gray R. S. Griffin J. Burstein P. Insel J. Scarlett J. A.. (1981). Receptor and postreceptor defects contribute to the insulin resistance in noninsulin-dependent diabetes mellitus. J. Clin. Invest. 68, 957–969. doi: 10.1172/jci110350, PMID: 7287908 PMC370882

[ref65] Kong L. Zhao Q. Jiang X. Hu J. Jiang Q. Sheng L.. (2024). Trimethylamine N-oxide impairs β-cell function and glucose tolerance. Nat. Commun. 15:2526. doi: 10.1038/s41467-024-46829-0, PMID: 38514666 PMC10957989

[ref66] Le Chatelier E. Nielsen T. Qin J. Prifti E. Hildebrand F. Falony G.. (2013). Richness of human gut microbiome correlates with metabolic markers. Nature 500, 541–546. doi: 10.1038/nature12506, PMID: 23985870

[ref67] Lee H. An J. Kim J. Choi D. Song Y. Lee C.-K.. (2022). A novel bacterium, *Butyricimonas virosa*, preventing HFD-induced diabetes and metabolic disorders in mice via GLP-1 receptor. Front. Microbiol. 13:858192. doi: 10.3389/fmicb.2022.858192, PMID: 35655996 PMC9152154

[ref68] Lee Y. Lee H.-Y. (2020). Revisiting the bacterial phylum composition in metabolic diseases focused on host energy metabolism. Diabetes Metab. J. 44, 658–667. doi: 10.4093/dmj.2019.0220, PMID: 32662252 PMC7643595

[ref69] Lee Y.-S. Lee D. Park G.-S. Ko S.-H. Park J. Lee Y.-K.. (2021). *Lactobacillus plantarum* HAC01 ameliorates type 2 diabetes in high-fat diet and streptozotocin-induced diabetic mice in association with modulating the gut microbiota. Food Funct. 12, 6363–6373. doi: 10.1039/d1fo00698c, PMID: 34105563

[ref70] Lefebvre P. Cariou B. Lien F. Kuipers F. Staels B. (2009). Role of bile acids and bile acid receptors in metabolic regulation. Physiol. Rev. 89, 147–191. doi: 10.1152/physrev.00010.2008, PMID: 19126757

[ref71] Li S.-Y. Chen S. Lu X.-T. Fang A.-P. Chen Y.-M. Huang R.-Z.. (2022). Serum trimethylamine-N-oxide is associated with incident type 2 diabetes in middle-aged and older adults: a prospective cohort study. J. Transl. Med. 20:374. doi: 10.1186/s12967-022-03581-7, PMID: 35982495 PMC9389664

[ref72] Li Q. Hu J. Nie Q. Chang X. Fang Q. Xie J.. (2021). Hypoglycemic mechanism of polysaccharide from Cyclocarya paliurus leaves in type 2 diabetic rats by gut microbiota and host metabolism alteration. Sci. China Life Sci. 64, 117–132. doi: 10.1007/s11427-019-1647-6, PMID: 32562054

[ref73] Li A. Lin C. Xie F. Jin M. Lin F. (2022). Berberine ameliorates insulin resistance by inhibiting IKK/NF-κB, JNK, and IRS-1/AKT signaling pathway in liver of gestational diabetes mellitus rats. Metab. Syndr. Relat. Disord. 20, 480–488. doi: 10.1089/met.2022.0017, PMID: 35862014

[ref74] Li X. Wang N. Yin B. Fang D. Jiang T. Fang S.. (2016). Effects of *Lactobacillus plantarum* CCFM0236 on hyperglycaemia and insulin resistance in high-fat and streptozotocin-induced type 2 diabetic mice. J. Appl. Microbiol. 121, 1727–1736. doi: 10.1111/jam.13276, PMID: 27552342

[ref75] Li W. Zhuang T. Wang Z. Wang X. Liu L. Luo Y.. (2023). Red ginseng extracts ameliorate high-fat diet-induced obesity and insulin resistance by activating the intestinal TGR5-mediated bile acids signaling pathway. Phytomed. Int. J. Phytother. Phytopharm. 119:154982. doi: 10.1016/j.phymed.2023.154982, PMID: 37531904

[ref76] Lian F. Li G. Chen X. Wang X. Piao C. Wang J.. (2014). Chinese herbal medicine Tianqi reduces progression from impaired glucose tolerance to diabetes: a double-blind, randomized, placebo-controlled, multicenter trial. J. Clin. Endocrinol. Metab. 99, 648–655. doi: 10.1210/jc.2013-3276, PMID: 24432995

[ref77] Liang Y.-C. Li L. Liang J.-L. Liu D.-L. Chu S.-F. Li H.-L. (2024). Integrating Mendelian randomization and single-cell RNA sequencing to identify therapeutic targets of baicalin for type 2 diabetes mellitus. Front. Pharmacol. 15:1403943. doi: 10.3389/fphar.2024.1403943, PMID: 39130628 PMC11310057

[ref78] Liang Y. Luo S. Cincotta A. H. (1999). Long-term infusion of norepinephrine plus serotonin into the ventromedial hypothalamus impairs pancreatic islet function. Metabolism 48, 1287–1289. doi: 10.1016/s0026-0495(99)90269-x, PMID: 10535392

[ref79] Liu W.-C. Chen P.-H. Chen L.-W. (2020). Supplementation of endogenous Ahr ligands reverses insulin resistance and associated inflammation in an insulin-dependent diabetic mouse model. J. Nutr. Biochem. 83:108384. doi: 10.1016/j.jnutbio.2020.108384, PMID: 32512500

[ref80] Liu G. Li Y. Pan A. Hu Y. Chen S. Qian F.. (2023). Adherence to a healthy lifestyle in association with microvascular complications among adults with type 2 diabetes. JAMA Netw. Open 6:e2252239. doi: 10.1001/jamanetworkopen.2022.52239, PMID: 36701156 PMC9880795

[ref81] Liu R. Wang J. Zhao Y. Zhou Q. Yang X. Gao Y.. (2024). Study on the mechanism of modified Gegen Qinlian decoction in regulating the intestinal flora-bile acid-TGR5 axis for the treatment of type 2 diabetes mellitus based on macro genome sequencing and targeted metabonomics integration. Phytomed. Int. J. Phytother. Phytopharm. 132:155329. doi: 10.1016/j.phymed.2023.155329, PMID: 38853123

[ref82] Liu H. Zhang M. Ma Q. Tian B. Nie C. Chen Z.. (2020). Health beneficial effects of resistant starch on diabetes and obesity via regulation of gut microbiota: a review. Food Funct. 11, 5749–5767. doi: 10.1039/d0fo00855a, PMID: 32602874

[ref83] Lu J. Gao Y. Gong Y. Yue Y. Yang Y. Xiong Y.. (2024). *Lycium barbarum* L. balanced intestinal flora with YAP1/FXR activation in drug-induced liver injury. Int. Immunopharmacol. 130:111762. doi: 10.1016/j.intimp.2024.111762, PMID: 38428146

[ref84] Lu J. Gong Y. Gao Y. Yang Y. Zhang Y. Zhang Z.. (2023). Wolfberry, yam, and Chrysanthemum polysaccharides increased intestinal *Akkermansia muciniphila* abundance and hepatic YAP1 expression to alleviate DILI. FASEB J. Off. Publ. Fed. Am. Soc. Exp. Biol. 37:e23286. doi: 10.1096/fj.202301388R 37950623

[ref85] Luo S. Luo J. Cincotta A. H. (1999). Chronic ventromedial hypothalamic infusion of norepinephrine and serotonin promotes insulin resistance and glucose intolerance. Neuroendocrinology 70, 460–465. doi: 10.1159/000054508, PMID: 10657739

[ref86] Luo Z. Xu J. Gao Q. Wang Z. Hou M. Liu Y. (2023). Study on the effect of licochalcone a on intestinal flora in type 2 diabetes mellitus mice based on 16S rRNA technology. Food Funct. 14, 8903–8921. doi: 10.1039/d3fo00861d, PMID: 37702574

[ref87] Luo Z. Xu W. Zhang Y. Di L. Shan J. (2020). A review of saponin intervention in metabolic syndrome suggests further study on intestinal microbiota. Pharmacol. Res. 160:105088. doi: 10.1016/j.phrs.2020.105088, PMID: 32683035

[ref88] Ma X. Qiu Y. Mao M. Lu B. Zhao H. Pang Z.. (2024). PuRenDan alleviates type 2 diabetes mellitus symptoms by modulating the gut microbiota and its metabolites. J. Ethnopharmacol. 322:117627. doi: 10.1016/j.jep.2023.117627 38147943

[ref89] Macfarlane S. Macfarlane G. T. (2003). Regulation of short-chain fatty acid production. Proc. Nutr. Soc. 62, 67–72. doi: 10.1079/PNS2002207, PMID: 12740060

[ref90] Magkos F. Hjorth M. F. Astrup A. (2020). Diet and exercise in the prevention and treatment of type 2 diabetes mellitus. Nat. Rev. Endocrinol. 16, 545–555. doi: 10.1038/s41574-020-0381-5 32690918

[ref91] Miao J. Ling A. V. Manthena P. V. Gearing M. E. Graham M. J. Crooke R. M.. (2015). Flavin-containing monooxygenase 3 as a potential player in diabetes-associated atherosclerosis. Nat. Commun. 6:6498. doi: 10.1038/ncomms7498, PMID: 25849138 PMC4391288

[ref9002] Mirjalili M. Salari Sharif A. Sangouni A. A. Emtiazi H. Mozaffari-Khosravi H. (2023). Effect of probiotic yogurt consumption on glycemic control and lipid profile in patients with type 2 diabetes mellitus: A randomized controlled trial. Clin. Nutr. ESPEN 54, 144–149. doi: 10.1016/j.clnesp.2023.01.014 36963856

[ref93] Moroti C. Souza Magri L. F. de Rezende Costa M. Cavallini D. C. Sivieri K. (2012). Effect of the consumption of a new symbiotic shake on glycemia and cholesterol levels in elderly people with type 2 diabetes mellitus. Lipids Health Dis. 11:29. doi: 10.1186/1476-511X-11-29, PMID: 22356933 PMC3305430

[ref94] Morrison D. J. Preston T. (2016). Formation of short chain fatty acids by the gut microbiota and their impact on human metabolism. Gut Microbes 7, 189–200. doi: 10.1080/19490976.2015.1134082, PMID: 26963409 PMC4939913

[ref95] Narang A. Rashid M. Thakur S. Jain S. K. Kaur A. Kaur S. (2024). Acute pre- and post-administration of Lactiplantibacillus plantarum 2034 and its secretory metabolites ameliorates Hyperglycaemia, Hyperlipidaemia, and oxidative stress in diabetic rats. Probiot. Antimicrob. Proteins. doi: 10.1007/s12602-024-10343-y 39150651

[ref96] Ng S. C. Xu Z. Mak J. W. Y. Yang K. Liu Q. Zuo T.. (2022). Microbiota engraftment after faecal microbiota transplantation in obese subjects with type 2 diabetes: a 24-week, double-blind, randomised controlled trial. Gut 71, 716–723. doi: 10.1136/gutjnl-2020-323617, PMID: 33785557

[ref97] Ojo O. Feng Q.-Q. Ojo O. O. Wang X.-H. (2020). The role of dietary fibre in modulating gut microbiota Dysbiosis in patients with type 2 diabetes: a systematic review and Meta-analysis of randomised controlled trials. Nutrients 12:3239. doi: 10.3390/nu12113239, PMID: 33113929 PMC7690692

[ref98] Olefsky J. M. Glass C. K. (2010). Macrophages, inflammation, and insulin resistance. Annu. Rev. Physiol. 72, 219–246. doi: 10.1146/annurev-physiol-021909-135846 20148674

[ref99] Palacios T. Vitetta L. Coulson S. Madigan C. D. Lam Y. Y. Manuel R.. (2020). Targeting the intestinal microbiota to prevent type 2 diabetes and enhance the effect of metformin on glycaemia: a randomised controlled pilot study. Nutrients 12:2041. doi: 10.3390/nu12072041, PMID: 32660025 PMC7400852

[ref100] Pedro M. N. Magro D. O. da Silva E. U. P. P. Guadagnini D. Santos A. de Jesus Pedro R.. (2018). Plasma levels of lipopolysaccharide correlate with insulin resistance in HIV patients. Diabetol. Metab. Syndr. 10:5. doi: 10.1186/s13098-018-0308-7, PMID: 29434676 PMC5793397

[ref101] Perino A. Pols T. W. H. Nomura M. Stein S. Pellicciari R. Schoonjans K. (2014). TGR5 reduces macrophage migration through mTOR-induced C/EBPβ differential translation. J. Clin. Invest. 124, 5424–5436. doi: 10.1172/JCI76289, PMID: 25365223 PMC4348975

[ref102] Petersen M. C. Shulman G. I. (2018). Mechanisms of insulin action and insulin resistance. Physiol. Rev. 98, 2133–2223. doi: 10.1152/physrev.00063.2017, PMID: 30067154 PMC6170977

[ref103] Pham N. H. T. Joglekar M. V. Wong W. K. M. Nassif N. T. Simpson A. M. Hardikar A. A. (2024). Short-chain fatty acids and insulin sensitivity: a systematic review and meta-analysis. Nutr. Rev. 82, 193–209. doi: 10.1093/nutrit/nuad042, PMID: 37290429 PMC10777678

[ref104] Qi Q. Li J. Yu B. Moon J.-Y. Chai J. C. Merino J.. (2022). Host and gut microbial tryptophan metabolism and type 2 diabetes: an integrative analysis of host genetics, diet, gut microbiome and circulating metabolites in cohort studies. Gut 71, 1095–1105. doi: 10.1136/gutjnl-2021-324053, PMID: 34127525 PMC8697256

[ref105] Qian X. Si Q. Lin G. Zhu M. Lu J. Zhang H.. (2022). *Bifidobacterium adolescentis* is effective in relieving type 2 diabetes and may be related to its dominant Core genome and gut microbiota modulation capacity. Nutrients 14:2479. doi: 10.3390/nu14122479, PMID: 35745208 PMC9227778

[ref106] Qin J. Li Y. Cai Z. Li S. Zhu J. Zhang F.. (2012). A metagenome-wide association study of gut microbiota in type 2 diabetes. Nature 490, 55–60. doi: 10.1038/nature11450, PMID: 23023125

[ref107] Ridaura V. K. Faith J. J. Rey F. E. Cheng J. Duncan A. E. Kau A. L.. (2013). Gut microbiota from twins discordant for obesity modulate metabolism in mice. Science 341:1241214. doi: 10.1126/science.1241214, PMID: 24009397 PMC3829625

[ref108] Rinninella E. Raoul P. Cintoni M. Franceschi F. Miggiano G. A. D. Gasbarrini A.. (2019). What is the healthy gut microbiota composition? A changing ecosystem across age, environment, diet, and diseases. Microorganisms 7:14. doi: 10.3390/microorganisms7010014, PMID: 30634578 PMC6351938

[ref109] Ruppin H. Bar-Meir S. Soergel K. H. Wood C. M. Schmitt M. G. (1980). Absorption of short-chain fatty acids by the colon. Gastroenterology 78, 1500–1507. doi: 10.1016/S0016-5085(19)30508-6 6768637

[ref110] Ryuk J. A. Lixia M. Cao S. Ko B.-S. Park S. (2017). Efficacy and safety of Gegen Qinlian decoction for normalizing hyperglycemia in diabetic patients: a systematic review and meta-analysis of randomized clinical trials. Complement. Ther. Med. 33, 6–13. doi: 10.1016/j.ctim.2017.05.004, PMID: 28735827

[ref111] Samokhvalov V. Bilan P. J. Schertzer J. D. Antonescu C. N. Klip A. (2009). Palmitate- and lipopolysaccharide-activated macrophages evoke contrasting insulin responses in muscle cells. Am. J. Physiol. Endocrinol. Metab. 296, E37–E46. doi: 10.1152/ajpendo.90667.2008, PMID: 18840759

[ref112] Samuel V. T. Shulman G. I. (2012). Mechanisms for insulin resistance: common threads and missing links. Cell 148, 852–871. doi: 10.1016/j.cell.2012.02.017, PMID: 22385956 PMC3294420

[ref113] Scheithauer T. P. M. Rampanelli E. Nieuwdorp M. Vallance B. A. Verchere C. B. Van Raalte D. H.. (2020). Gut microbiota as a trigger for metabolic inflammation in obesity and type 2 diabetes. Front. Immunol. 11:571731. doi: 10.3389/fimmu.2020.571731, PMID: 33178196 PMC7596417

[ref114] Schlienger J.-L. (2013). Type 2 diabetes complications. Presse Med. 42, 839–848. doi: 10.1016/j.lpm.2013.02.313, PMID: 23528336

[ref115] Schultze S. M. Hemmings B. A. Niessen M. Tschopp O. (2012). PI3K/AKT, MAPK and AMPK signalling: protein kinases in glucose homeostasis. Expert Rev. Mol. Med. 14:e1. doi: 10.1017/S1462399411002109, PMID: 22233681

[ref116] Sender R. Fuchs S. Milo R. (2016). Revised estimates for the number of human and Bacteria cells in the body. PLoS Biol. 14:e1002533. doi: 10.1371/journal.pbio.1002533, PMID: 27541692 PMC4991899

[ref117] Shao C. Sun M. Liu W. Zhao S. Liu Y. Chen Y.. (2022). Patient-reported outcomes following the use of Jiang Tang san Huang tablets in type 2 diabetes mellitus: a retrospective cohort study in a Chinese population. Diab. Metab. Syndr. Obes. Targets Ther. 15, 4023–4033. doi: 10.2147/DMSO.S388336, PMID: 36582504 PMC9793732

[ref118] She J. Tuerhongjiang G. Guo M. Liu J. Hao X. Guo L.. (2024). Statins aggravate insulin resistance through reduced blood glucagon-like peptide-1 levels in a microbiota-dependent manner. Cell Metab. 36, 408–421.e5. doi: 10.1016/j.cmet.2023.12.027, PMID: 38325336

[ref119] Shoelson S. E. Lee J. Goldfine A. B. (2006). Inflammation and insulin resistance. J. Clin. Invest. 116, 1793–1801. doi: 10.1172/JCI29069, PMID: 16823477 PMC1483173

[ref120] Song Q. Cheng S. W. Li D. Cheng H. Lai Y. S. Han Q.. (2022). Gut microbiota mediated hypoglycemic effect of Astragalus membranaceus polysaccharides in db/db mice. Front. Pharmacol. 13:1043527. doi: 10.3389/fphar.2022.1043527, PMID: 36452223 PMC9703139

[ref121] Su J. Luo Y. Hu S. Tang L. Ouyang S. (2023). Advances in research on type 2 diabetes mellitus targets and therapeutic agents. Int. J. Mol. Sci. 24:13381. doi: 10.3390/ijms241713381, PMID: 37686185 PMC10487533

[ref122] Sun J. Ren J. Hu X. Hou Y. Yang Y. (2021). Therapeutic effects of Chinese herbal medicines and their extracts on diabetes. Biomed. Pharmacother. 142:111977. doi: 10.1016/j.biopha.2021.111977, PMID: 34364042

[ref123] Sun W. Zhang D. Wang Z. Sun J. Xu B. Chen Y.. (2016). Insulin resistance is associated with Total bile acid level in type 2 diabetic and nondiabetic population: a cross-sectional study. Medicine (Baltimore) 95:e2778. doi: 10.1097/MD.0000000000002778, PMID: 26962776 PMC4998857

[ref124] Sun L. Zhang X. Zhang Y. Zheng K. Xiang Q. Chen N.. (2019). Antibiotic-induced disruption of gut microbiota alters local metabolomes and immune responses. Front. Cell. Infect. Microbiol. 9:99. doi: 10.3389/fcimb.2019.00099, PMID: 31069173 PMC6491449

[ref125] Takeuchi T. Kubota T. Nakanishi Y. Tsugawa H. Suda W. Kwon A. T.-J.. (2023). Gut microbial carbohydrate metabolism contributes to insulin resistance. Nature 621, 389–395. doi: 10.1038/s41586-023-06466-x, PMID: 37648852 PMC10499599

[ref126] Tan Y. Liu S. Huang M. Cheng H. Xu B. Luo H.. (2023). Efficacy and safety of Gegen Qinlian decoction in the treatment of type II diabetes mellitus: a systematic review and meta-analysis of randomized clinical trials. Front. Endocrinol. 14:1316269. doi: 10.3389/fendo.2023.1316269, PMID: 38344688 PMC10858613

[ref127] Tang C. Ahmed K. Gille A. Lu S. Gröne H.-J. Tunaru S.. (2015). Loss of FFA2 and FFA3 increases insulin secretion and improves glucose tolerance in type 2 diabetes. Nat. Med. 21, 173–177. doi: 10.1038/nm.3779, PMID: 25581519

[ref128] Tawulie D. Jin L. Shang X. Li Y. Sun L. Xie H.. (2023). Jiang-Tang-san-Huang pill alleviates type 2 diabetes mellitus through modulating the gut microbiota and bile acids metabolism. Phytomed. Int. J. Phytother. Phytopharm. 113:154733. doi: 10.1016/j.phymed.2023.154733, PMID: 36870307

[ref129] Tazoe H. Otomo Y. Karaki S.-I. Kato I. Fukami Y. Terasaki M.. (2009). Expression of short-chain fatty acid receptor GPR41 in the human colon. Biomed. Res. Tokyo Jpn. 30, 149–156. doi: 10.2220/biomedres.30.149 19574715

[ref130] Tian J. Bai B. Gao Z. Yang Y. Wu H. Wang X.. (2021). Alleviation effects of GQD, a traditional Chinese medicine formula, on diabetes rats linked to modulation of the gut microbiome. Front. Cell. Infect. Microbiol. 11:740236. doi: 10.3389/fcimb.2021.740236, PMID: 34692563 PMC8531589

[ref131] Toda G. Soeda K. Okazaki Y. Kobayashi N. Masuda Y. Arakawa N.. (2020). Insulin- and lipopolysaccharide-mediated signaling in adipose tissue macrophages regulates postprandial Glycemia through Akt-mTOR activation. Mol. Cell 79, 43–53.e4. doi: 10.1016/j.molcel.2020.04.033, PMID: 32464093 PMC11969070

[ref132] Tolhurst G. Heffron H. Lam Y. S. Parker H. E. Habib A. M. Diakogiannaki E.. (2012). Short-chain fatty acids stimulate glucagon-like peptide-1 secretion via the G-protein-coupled receptor FFAR2. Diabetes 61, 364–371. doi: 10.2337/db11-1019, PMID: 22190648 PMC3266401

[ref133] Tong X. Xu J. Lian F. Yu X. Zhao Y. Xu L.. (2018). Structural alteration of gut microbiota during the amelioration of human type 2 diabetes with hyperlipidemia by metformin and a traditional Chinese herbal formula: a multicenter, randomized, open label clinical trial. MBio 9, e02392–e02317. doi: 10.1128/mBio.02392-17, PMID: 29789365 PMC5964358

[ref9003] Tonucci L. B. Olbrich Dos Santos K. M. Licursi de Oliveira L. Rocha Ribeiro S. M. Duarte Martino H. S. (2017). Clinical application of probiotics in type 2 diabetes mellitus: A randomized, double-blind, placebo-controlled study. Clin. Nutr. Edinb. Scotl. 36, 85–92. doi: 10.1016/j.clnu.2015.11.011 26732026

[ref134] Venkatesh M. Mukherjee S. Wang H. Li H. Sun K. Benechet A. P.. (2014). Symbiotic bacterial metabolites regulate gastrointestinal barrier function via the xenobiotic sensor PXR and toll-like receptor 4. Immunity 41, 296–310. doi: 10.1016/j.immuni.2014.06.014, PMID: 25065623 PMC4142105

[ref135] Verbrugge F. H. (2017). Role of SGLT2 inhibitors in patients with diabetes mellitus and heart failure. Curr. Heart Fail. Rep. 14, 275–283. doi: 10.1007/s11897-017-0340-1, PMID: 28647919

[ref136] Wahlström A. Sayin S. I. Marschall H.-U. Bäckhed F. (2016). Intestinal crosstalk between bile acids and microbiota and its impact on host metabolism. Cell Metab. 24, 41–50. doi: 10.1016/j.cmet.2016.05.005, PMID: 27320064

[ref137] Wan J. An L. Ren Z. Wang S. Yang H. Ma J. (2023). Effects of galactooligosaccharides on maternal gut microbiota, glucose metabolism, lipid metabolism and inflammation in pregnancy: a randomized controlled pilot study. Front. Endocrinol. 14:1034266. doi: 10.3389/fendo.2023.1034266 PMC9911812 36777355

[ref138] Wang H. Guo L. Shang H. Ren M. Wang X. Wang D.. (2017). JinqiJiangtang tablets for pre-diabetes: a randomized, double-blind and placebo-controlled clinical trial. Sci. Rep. 7:11190. doi: 10.1038/s41598-017-11583-5 28894283 PMC5593818

[ref139] Wang Y. Liu H. Zheng M. Yang Y. Ren H. Kong Y.. (2021). Berberine slows the progression of prediabetes to diabetes in Zucker diabetic fatty rats by enhancing intestinal secretion of glucagon-like Peptide-2 and improving the gut microbiota. Front. Endocrinol. 12:609134. doi: 10.3389/fendo.2021.609134, PMID: 34025574 PMC8138858

[ref140] Wang J. Ma Q. Li Y. Li P. Wang M. Wang T.. (2020). Research progress on traditional Chinese medicine syndromes of diabetes mellitus. Biomed. Pharmacother. Biomed. Pharmacother. 121:109565. doi: 10.1016/j.biopha.2019.109565, PMID: 31704615

[ref141] Wang Z. Wang X. Fu L. Xu S. Wang X. Liao Q.. (2024). Shengmai san formula alleviates high-fat diet-induced obesity in mice through gut microbiota-derived bile acid promotion of M2 macrophage polarization and thermogenesis. Phytomed. Int. J. Phytother. Phytopharm. 133:155938. doi: 10.1016/j.phymed.2024.155938, PMID: 39163753

[ref142] Wang X.-H. Xu F. Cheng M. Wang X. Zhang D.-M. Zhao L.-H.. (2020). Fasting serum total bile acid levels are associated with insulin sensitivity, islet β-cell function and glucagon levels in response to glucose challenge in patients with type 2 diabetes. Endocr. J. 67, 1107–1117. doi: 10.1507/endocrj.EJ20-0201, PMID: 32684527

[ref143] Wijdeveld M. Schrantee A. Hagemeijer A. Nederveen A. J. Scheithauer T. P. M. Levels J. H. M.. (2023). Intestinal acetate and butyrate availability is associated with glucose metabolism in healthy individuals. iScience 26:108478. doi: 10.1016/j.isci.2023.108478, PMID: 38094244 PMC10716539

[ref144] Won G. Choi S.-I. Kang C.-H. Kim G.-H. (2021). Lactiplantibacillus plantarum MG4296 and Lacticaseibacillus paracasei MG5012 ameliorates insulin resistance in palmitic acid-induced HepG2 cells and high fat diet-induced mice. Microorganisms 9:1139. doi: 10.3390/microorganisms9061139, PMID: 34070604 PMC8228052

[ref145] Wu J. Yang K. Fan H. Wei M. Xiong Q. (2023). Targeting the gut microbiota and its metabolites for type 2 diabetes mellitus. Front. Endocrinol. 14:1114424. doi: 10.3389/fendo.2023.1114424, PMID: 37229456 PMC10204722

[ref146] Wu Z. Zhang B. Chen F. Xia R. Zhu D. Chen B.. (2022). Fecal microbiota transplantation reverses insulin resistance in type 2 diabetes: a randomized, controlled, prospective study. Front. Cell. Infect. Microbiol. 12:1089991. doi: 10.3389/fcimb.2022.1089991, PMID: 36704100 PMC9872724

[ref147] Xia T. Liu C.-S. Hu Y.-N. Luo Z.-Y. Chen F.-L. Yuan L.-X.. (2021). Coix seed polysaccharides alleviate type 2 diabetes mellitus via gut microbiota-derived short-chain fatty acids activation of IGF1/PI3K/AKT signaling. Food Res. Int. 150:110717. doi: 10.1016/j.foodres.2021.110717, PMID: 34865748

[ref148] Xiao R. Wang L. Tian P. Jin X. Zhao J. Zhang H.. (2023). The effect of probiotic supplementation on glucolipid metabolism in patients with type 2 diabetes: a systematic review and Meta-analysis. Nutrients 15:3240. doi: 10.3390/nu15143240, PMID: 37513657 PMC10383415

[ref149] Xu X. Gao Z. Yang F. Yang Y. Chen L. Han L.. (2020). Antidiabetic effects of Gegen Qinlian decoction via the gut microbiota are attributable to its key ingredient Berberine. Genomics Proteomics Bioinformatics 18, 721–736. doi: 10.1016/j.gpb.2019.09.007, PMID: 33359679 PMC8377040

[ref150] Xu X. Hui H. Cai D. (2012). Differences in fecal Bifidobacterium species between patients with type 2 diabetes and healthy individuals. Nan Fang Yi Ke Da Xue Xue Bao 32:564. 22543136

[ref151] Xu C.-X. Wang C. Zhang Z.-M. Jaeger C. D. Krager S. L. Bottum K. M.. (2015). Aryl hydrocarbon receptor deficiency protects mice from diet-induced adiposity and metabolic disorders through increased energy expenditure. Int. J. Obes. 39, 1300–1309. doi: 10.1038/ijo.2015.63, PMID: 25907315 PMC4526411

[ref152] Xu Y. Zheng S. Jiang S. Chen J. Zhu X. Zhang Y. (2022). The effect of Chinese herbal formulas combined with metformin on modulating the gut microbiota in the amelioration of type 2 diabetes mellitus: a systematic review and meta-analysis. Front. Endocrinol. 13:927959. doi: 10.3389/fendo.2022.927959, PMID: 36187136 PMC9521410

[ref153] Yabut J. M. Desjardins E. M. Chan E. J. Day E. A. Leroux J. M. Wang B.. (2020). Genetic deletion of mast cell serotonin synthesis prevents the development of obesity and insulin resistance. Nat. Commun. 11:463. doi: 10.1038/s41467-019-14080-7, PMID: 31974364 PMC6978527

[ref154] Yan X. Zhang Y. Peng Y. Li X. (2022). The water extract of Radix scutellariae, its total flavonoids and baicalin inhibited CYP7A1 expression, improved bile acid, and glycolipid metabolism in T2DM mice. J. Ethnopharmacol. 293:115238. doi: 10.1016/j.jep.2022.115238, PMID: 35351576

[ref155] Yang Y. Chang Y. Wu Y. Liu H. Liu Q. Kang Z.. (2021). A homogeneous polysaccharide from *Lycium barbarum*: structural characterizations, anti-obesity effects and impacts on gut microbiota. Int. J. Biol. Macromol. 183, 2074–2087. doi: 10.1016/j.ijbiomac.2021.05.209, PMID: 34097961

[ref156] Yang X. Dong B. An L. Zhang Q. Chen Y. Wang H.. (2021). Ginsenoside Rb1 ameliorates glycemic disorder in mice with high fat diet-induced obesity via regulating gut microbiota and amino acid metabolism. Front. Pharmacol. 12:756491. doi: 10.3389/fphar.2021.756491, PMID: 34899310 PMC8654325

[ref157] Yao B. Pan B. Tian T. Su X. Zhang S. Li H.. (2022). Baihu renshen decoction ameliorates type 2 diabetes mellitus in rats through affecting gut microbiota enhancing gut permeability and inhibiting TLR4/NF-κB-mediated inflammatory response. Front. Cell. Infect. Microbiol. 12:1051962. doi: 10.3389/fcimb.2022.1051962, PMID: 36439213 PMC9691847

[ref158] Yao Y. Yan L. Chen H. Wu N. Wang W. Wang D. (2020). Cyclocarya paliurus polysaccharides alleviate type 2 diabetic symptoms by modulating gut microbiota and short-chain fatty acids. Phytomed. Int. J. Phytother. Phytopharm. 77:153268. doi: 10.1016/j.phymed.2020.153268, PMID: 32663709

[ref159] Zaharieva D. P. McGaugh S. Davis E. A. Riddell M. C. (2020). Advances in exercise, physical activity, and diabetes. Diabetes Technol. Ther. 22, S-109–S-118. doi: 10.1089/dia.2020.2508, PMID: 32069147

[ref160] Zeng S.-Y. Liu Y.-F. Zeng Z.-L. Zhao Z.-B. Yan X.-L. Zheng J.. (2024). Antibiotic-induced gut microbiota disruption promotes vascular calcification by reducing short-chain fatty acid acetate. Mol. Med. 30:130. doi: 10.1186/s10020-024-00900-0, PMID: 39182021 PMC11344439

[ref161] Zhang P. (2022). Influence of foods and nutrition on the gut microbiome and implications for intestinal health. Int. J. Mol. Sci. 23:9588. doi: 10.3390/ijms23179588, PMID: 36076980 PMC9455721

[ref162] Zhang Y. Gu Y. Ren H. Wang S. Zhong H. Zhao X.. (2020). Gut microbiome-related effects of berberine and probiotics on type 2 diabetes (the PREMOTE study). Nat. Commun. 11:5015. doi: 10.1038/s41467-020-18414-8, PMID: 33024120 PMC7538905

[ref163] Zhang Z. He Y. Zhao M. He X. Zhou Z. Yue Y.. (2024). Qinlian Hongqu decoction modulates FXR/TGR5/GLP-1 pathway to improve insulin resistance in NAFLD mice: bioinformatics and experimental study. ACS Omega 9, 45447–45466. doi: 10.1021/acsomega.4c07463, PMID: 39554433 PMC11561767

[ref164] Zhang P.-P. Li L.-L. Han X. Li Q.-W. Zhang X.-H. Liu J. J.. (2020). Fecal microbiota transplantation improves metabolism and gut microbiome composition in db/db mice. Acta Pharmacol. Sin. 41, 678–685. doi: 10.1038/s41401-019-0330-9, PMID: 31937933 PMC7468362

[ref165] Zhang H. Ma L. Peng W. Wang B. Sun Y. (2024). Association between gut microbiota and onset of type 2 diabetes mellitus: a two-sample Mendelian randomization study. Front. Cell. Infect. Microbiol. 14:1327032. doi: 10.3389/fcimb.2024.1327032, PMID: 38596649 PMC11002178

[ref166] Zhang X. Zhao Y. Xu J. Xue Z. Zhang M. Pang X.. (2015). Modulation of gut microbiota by berberine and metformin during the treatment of high-fat diet-induced obesity in rats. Sci. Rep. 5:14405. doi: 10.1038/srep14405, PMID: 26396057 PMC4585776

[ref167] Zhang X. Zhao Y. Zhang M. Pang X. Xu J. Kang C.. (2012). Structural changes of gut microbiota during berberine-mediated prevention of obesity and insulin resistance in high-fat diet-fed rats. PLoS One 7:e42529. doi: 10.1371/journal.pone.0042529, PMID: 22880019 PMC3411811

[ref168] Zhao J. Fang Z. (2024). Alterations of the gut microbiota and metabolites by ShenZhu TiaoPi granule alleviates hyperglycemia in GK rats. Front. Microbiol. 15:1420103. doi: 10.3389/fmicb.2024.1420103, PMID: 39372266 PMC11451479

[ref169] Zhao J. Li Y. Sun M. Xin L. Wang T. Wei L.. (2019). The Chinese herbal formula Shenzhu Tiaopi granule results in metabolic improvement in type 2 diabetic rats by modulating the gut microbiota. Evid. Based Complement. Altern. Med. 2019:6976394. doi: 10.1155/2019/6976394, PMID: 31275416 PMC6582833

[ref170] Zhao Z.-H. Xin F.-Z. Zhou D. Xue Y.-Q. Liu X.-L. Yang R.-X.. (2019). Trimethylamine N-oxide attenuates high-fat high-cholesterol diet-induced steatohepatitis by reducing hepatic cholesterol overload in rats. World J. Gastroenterol. 25, 2450–2462. doi: 10.3748/wjg.v25.i20.2450, PMID: 31171889 PMC6543245

[ref171] Zhao T. Yang M. Ma L. Liu X. Ding Q. Chai G.. (2023). Structural modification and biological activity of polysaccharides. Molecules 28:5416. doi: 10.3390/molecules28145416, PMID: 37513287 PMC10384959

[ref172] Zheng J. An Y. Du Y. Song Y. Zhao Q. Lu Y. (2024). Effects of short-chain fatty acids on blood glucose and lipid levels in mouse models of diabetes mellitus: a systematic review and network meta-analysis. Pharmacol. Res. 199:107041. doi: 10.1016/j.phrs.2023.107041, PMID: 38128856

[ref173] Zheng Y. Bai L. Zhou Y. Tong R. Zeng M. Li X.. (2019). Polysaccharides from Chinese herbal medicine for anti-diabetes recent advances. Int. J. Biol. Macromol. 121, 1240–1253. doi: 10.1016/j.ijbiomac.2018.10.072, PMID: 30342938

[ref174] Zhou R. He D. Zhang H. Xie J. Zhang S. Tian X.. (2023). Ginsenoside Rb1 protects against diabetes-associated metabolic disorders in Kkay mice by reshaping gut microbiota and fecal metabolic profiles. J. Ethnopharmacol. 303:115997. doi: 10.1016/j.jep.2022.115997, PMID: 36509256

[ref175] Zhu Q. An Y. A. Kim M. Zhang Z. Zhao S. Zhu Y.. (2020). Suppressing adipocyte inflammation promotes insulin resistance in mice. Mol. Metab. 39:101010. doi: 10.1016/j.molmet.2020.101010, PMID: 32408016 PMC7272509

[ref176] Zou H. Zhang M. Zhu X. Zhu L. Chen S. Luo M.. (2022). Ginsenoside Rb1 improves metabolic disorder in high-fat diet-induced obese mice associated with modulation of gut microbiota. Front. Microbiol. 13:826487. doi: 10.3389/fmicb.2022.826487, PMID: 35516426 PMC9062662

